# HJURP inhibits sensitivity to ferroptosis inducers in prostate cancer cells by enhancing the peroxidase activity of PRDX1

**DOI:** 10.1016/j.redox.2024.103392

**Published:** 2024-10-10

**Authors:** Wenjie Lai, Weian Zhu, Jianjie Wu, Jiongduan Huang, Xiaojuan Li, Yun Luo, Yu Wang, Hengda Zeng, Mingqiang Li, Xiaofu Qiu, Xingqiao Wen

**Affiliations:** a Department of Urology, The Affiliated Guangdong Second Provincial General Hospital of Jinan University, Guangzhou, Guangdong, 510317, PR China; b Department of Urology, The Third Affiliated Hospital, Sun Yat-sen University, Guangzhou, Guangdong, 510630, PR China; c Department of Urology, Shenshan Medical Center, Memorial Hospital of Sun Yat-sen University, Shanwei, Guangdong, 516600, PR China; d Department of Health Care, Shenzhen Hospital, Southern Medical University, Shenzhen, Guangdong, 518101, PR China; e Laboratory of Biomaterials and Translational Medicine, The Third Affiliated Hospital, Sun Yat-sen University, Guangzhou, Guangdong, 510630, PR China; f Department of Urology, Guangdong Provincial People's Hospital, Southern Medical University, Guangzhou, 510080, PR China

**Keywords:** Prostate cancer, Ferroptosis, HJURP, Peroxidase activity, Disulfide binding

## Abstract

Ferroptosis induction has emerged as a promising therapeutic approach for prostate cancer (PCa), either as a monotherapy or in combination with hormone therapy. Therefore, identifying the mechanisms regulating ferroptosis in PCa cells is essential. Our previous study demonstrated that HJURP, an oncogene upregulated in PCa cells, plays a role in tumor proliferation. Here, we expand these findings by elucidating a novel mechanism by which HJURP inhibits sensitivity to ferroptosis inducers in PCa cells via the PRDX1/reactive oxygen species (ROS) pathway *in vitro* and *in vivo*. Mechanistically, HJURP forms disulfide-linked intermediates with PRDX1 through Cys^327^ and Cys^457^ residues. This disulfide binding promotes PRDX1 redox cycling and inhibits its hyperoxidation. As a result, HJURP enhances the peroxidase activity of PRDX1, leading to a decrease in ROS levels and subsequently suppressing lipid peroxidation induced by ferroptosis inducers. These findings reveal the potential of HJURP/PRDX1 as novel therapeutic targets and biomarkers of ferroptosis in PCa patients.

## Highlights

• HJURP inhibits ferroptosis inducer-induced cell death in prostate cancer cells.
• HJURP suppresses ferroptosis via the PRDX1/ROS pathway.
• HJURP enhances PRDX1 peroxidase activity by forming disulfide bonds.
• HJURP forms disulfide-linked intermediates with PRDX1 through Cys327 and Cys457.

## Graphical abstract

**Figure:**
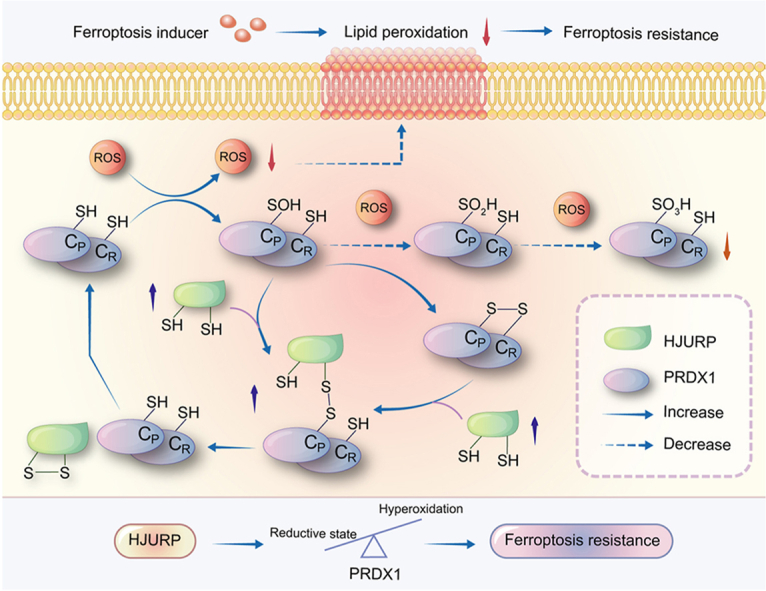

## 1 Introduction

Prostate cancer (PCa) represents a significant threat to male health, ranking as the second most common cancer and the fifth leading cause of cancer-related mortality in males worldwide [1,2]. In the early stages, radical prostatectomy and radiotherapy are the primary treatments for PCa [3]. However, a substantial proportion of patients (27–53 %) experience biochemical recurrence [4,5]. At an advanced stage, hormone therapies, including androgen deprivation therapy and anti-androgen therapy, have become mainstays for PCa patients [6]. Unfortunately, despite the initial response to hormone therapy, almost all patients develop treatment resistance, and the disease ultimately progresses to castration-resistant PCa [7], which accounts for the majority of PCa-related deaths. Therefore, there is an urgent need to explore new approaches for the treatment of PCa.

Ferroptosis is a novel form of regulated cell death characterized by iron overload, lipid peroxidation, and the accumulation of reactive oxygen species (ROS). Inducing ferroptosis has emerged as a promising therapeutic strategy for treating PCa [[8], [9], [10]]. Specifically, studies have shown that advanced PCa, such as castration-resistant PCa and neuroendocrine PCa, are particularly sensitive to ferroptosis inducers, which can induce cell death in these otherwise resistant forms of cancer [11,12]. These findings highlight the potential of targeting ferroptosis as a novel approach to combat PCa, underscoring the importance of investigating the mechanisms underlying ferroptosis in this disease.

Holliday junction recognition protein (HJURP), an oncogene involved in tumor progression and associated with poor prognosis, is aberrantly expressed in various tumors, including breast cancer, multiple myeloma, pancreatic cancer, and PCa [[13], [14], [15], [16]]. In our previous study [16], we observed that HJURP promotes PCa proliferation *in vitro* and *in vivo* by facilitating the ubiquitin-dependent proteasomal degradation of CDKN1A mediated via the GSK3β/JNK signaling pathway. In this study, we identified HJURP as a protein that influences the sensitivity of PCa cells to ferroptosis inducers. Specifically, through its Cys^327^ and Cys^457^ residues, HJURP forms disulfide-linked intermediates with peroxiredoxin 1 (PRDX1), effectively inhibiting the hyperoxidation of PRDX1. Moreover, the intermediates generated by this interaction can be transformed back into a reduced state and participate in subsequent redox cycles. Through this redox modification, HJURP upregulates the levels of reductive PRDX1 while downregulating the levels of hyperoxidized PRDX1, thereby enhancing the peroxidase activity of PRDX1, moderating ROS levels, and ultimately conferring resistance to ferroptosis inducers in PCa cells. Considering these findings, we confirmed that depletion of both HJURP and PRDX1 significantly enhances the sensitivity of PCa cells to ferroptosis inducers. Moreover, HJURP and PRDX1 expression were highly correlated in PCa tissues, and both proteins functioned as independent prognostic factors for overall survival (OS) in PCa patients.

In summary, our findings provide mechanistic insights into HJURP/PRDX1-mediated antioxidant defense and highlight the potential of HJURP/PRDX1 as novel diagnostic and therapeutic targets for ferroptosis-based treatment in PCa patients.

## 2 Results

### 2.1 HJURP inhibits the sensitivity of PCa cells to ferroptosis inducers

Our previous study showed that HJURP promotes the proliferation of PCa cells *in vitro* and *in vivo* [16]. To explore additional functions of HJURP in PCa cells, we generated HJURP-knockout C4-2 and PC3 cells, which had been previously shown to express the highest HJURP levels among the cells evaluated, using the CRISPR/Cas9 tool (Supplementary Fig. S1A). Subsequently, we evaluated the influence of HJURP knockout on various cellular processes, including ferroptosis, apoptosis, autophagy, necroptosis, migration and invasion, in PCa cells. The results showed that HJURP knockout significantly inhibited the migratory and invasive abilities of these PCa cells (Supplementary Figs. S1B–C) and enhanced their sensitivity to Erastin- or RSL3-induced ferroptosis (Fig. 1A and B, Supplementary Fig. S1D). However, HJURP knockout did not affect docetaxel-induced apoptosis (Supplementary Fig. S1E), rapamycin-induced autophagy (Supplementary Fig. S1F), or TNF-α-induced necroptosis (Supplementary Fig. S1G).

**Fig. 1:**
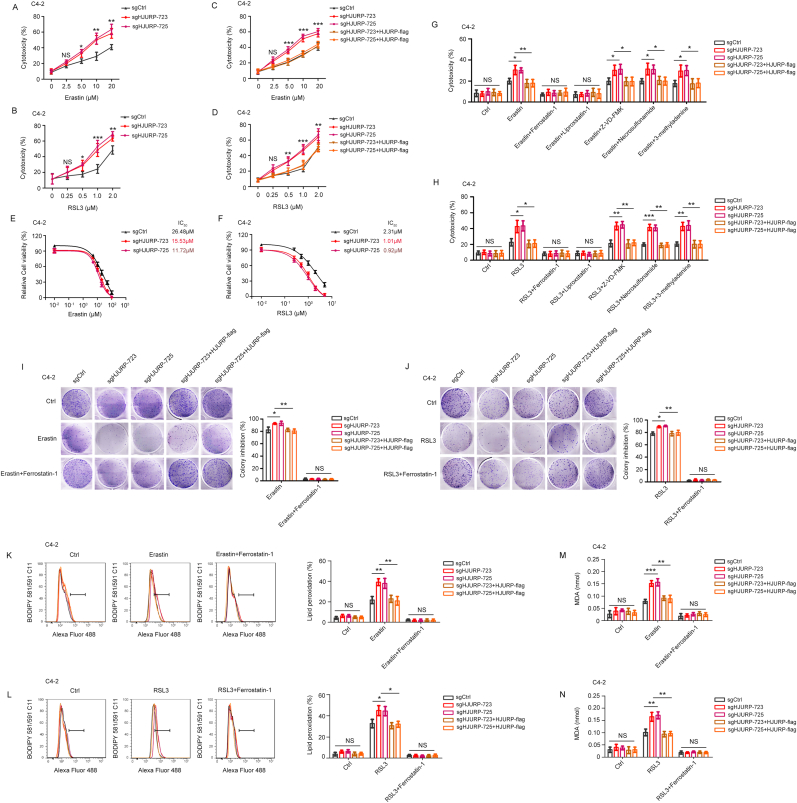
**HJURP inhibited PCa cells sensitivity to ferroptosis inducers. A-D.** Cytotoxicity assay of HJURP knockout/re-expressed C4-2 cells treated with indicated doses of Erastin (**A, C**) or RSL3 (**B, D**) for 24 h. **E-F.** 24-hour dose-response curves of HJURP knockout C4-2 cells with Erastin (**E**) or RSL3 (**F**) treatment. Relative cell viability was normalized to sgCtrl cells with Erastin (10^−2^ μM) or RSL3 (10^−2^ μM) treatment. **G-H.** 24-hour cytotoxicity assay of HJURP-regulated C4-2 cells treated with Erastin (5 μM, **G**) or RSL3 (1 μM, **H**) in the absence or presence of ferrostatin-1 (2 μM), liproxstatin-1 (1 μM), Z-VAD-FMK (10 μM), necrosulfonamide (0.5 μM), or 3-methyladenine (250 μM). **I-J.** Quantitative analysis of the effect of HJURP on clonogenic survival of C4-2 cells (2 × 10^3^ cells/well) treated with Erastin (3.5 μM), RSL3 (250 nM) or Ferrostatin-1 (1 μM) for 12 days. The colony number of the RSL3/Erastin or RSL3/Erastin + Ferrostatin-1 group in each HJURP-regulated group (sgCtrl. sgHJURP, etc.) was normalized to the colony number of its own Ctrl group. Colony inhibition (%) = 1-[colony number (RSL3/Erastin or RSL3/Erastin + Ferrostatin-1)/colony number (Ctrl)] × 100 %. **K–N.** HJURP suppressed lipid peroxidation (**K-L**) and MDA production (**M-N**) in C4-2 cells treated with/without Erastin (5 μM, 10 h), RSL3 (1 μM, 2 h), or ferrostatin-1 (2 μM, 24 h). 1-way ANOVA test was used to determine significance. Error bars indicate the SD from three independent experiments (Error bars for clonogenic survival assays were calculated from two independent experiments). ∗*P* < 0.05, ∗∗*P* < 0.01, ∗∗∗*P* < 0.001. IC_50_, half maximal inhibitory concentration.

Although the role of HJURP in regulating migration and invasion in other tumors has been reported [17], its involvement in the modulation of ferroptosis sensitivity in PCa cells remains poorly understood. Therefore, we aimed to elucidate the potential mechanism by which HJURP inhibits sensitivity to ferroptosis inducers in PCa cells. To further confirm the effect of HJURP on cell death induced by ferroptosis inducers, we rescued HJURP expression in knockout cells and observed a suppression of cell death (Fig. 1C and D, Supplementary Figs. S1H and S2A). Additionally, HJURP knockout PCa cells exhibited a lower half maximal inhibitory concentration (IC_50_) of Erastin and RSL3 (Fig. 1E and F, Supplementary Fig. S2B), further confirming the ability of HJURP to inhibit the sensitivity of PCa cells to ferroptosis inducers. Moreover, Erastin- or RSL3-induced cell death in PCa cells, regulated by HJURP, was significantly blocked by the ferroptosis inhibitors ferrostatin-1 and liproxstatin-1, but not by the apoptosis inhibitor Z-VAD-FMK, the necroptosis inhibitor necrosulfonamide, or the autophagy inhibitor 3-methyladenine (Fig. 1G and H, Supplementary Fig. S2C). Furthermore, HJURP knockout markedly enhanced Erastin- or RSL3-induced lipid peroxidation and cell colony inhibition, which was reversed after HJURP protein expression was rescued, and these effects were completely blocked by ferrostatin-1 (Fig. 1I–N, Supplementary Figs. S2D–F).

According to the aforementioned results, HJURP significantly suppresses the sensitivity of PCa cells to ferroptosis inducers.

### 2.2 HJURP suppresses sensitivity to ferroptosis inducers via the PRDX1/ROS pathway

To understand the mechanism by which HJURP inhibits cell death induced by ferroptosis inducers, we determined the main factors involved in ferroptosis modulation [18], including Fe^2+^ levels, GSH levels, ROS production, and the protein expression of xCT^−^, ACSL4 and GPX4, in HJURP-knockout PCa cells. The results showed that HJURP did not regulate the expression of these ferroptosis-related proteins (Supplementary Fig. S3A). However, HJURP knockout primarily increased ROS and Fe^2+^ levels, while decreasing GSH levels (Fig. 2A, Supplementary Figs. S3B–C). As the increase in ROS can promote the consumption of GSH [19] and the release of iron from heme-containing proteins or metal-binding proteins [20], we hypothesized that the changes in GSH and Fe^2+^ levels could be caused by the increase in ROS levels. Interestingly, the regulation of GSH and Fe^2+^ levels by HJURP disappeared when we cultured C4-2 and PC3 cells with the antioxidants N-acetyl-l-cysteine (NAC) or catechin hydrate (CH) (Supplementary Figs. S3B–C). Furthermore, the increased sensitivity of C4-2 and PC3 cells to RSL3 following HJURP knockout was abolished by catalase (Fig. 2B), a specific ROS scavenger, strongly suggesting that HJURP may suppress sensitivity to ferroptosis inducers by inhibiting ROS production. However, HJURP is not a peroxidase dedicated to ROS scavenging. Therefore, to determine the potential mechanism by which HJURP inhibits ROS production, we performed liquid chromatography-mass spectrometry (LC-MS/MS) analysis to identify the binding partners of HJURP. Among the putative binding peptides, the one belonging to PRDX1 and PRDX2 drew our attention, as the PRDXs family is the main antioxidant enzymes in eukaryotic cells [21] (Supplementary Fig. S3D). Next, Co-immunoprecipitation (Co-IP) assays showed that HJURP and PRDX1, but not PRDX2, reside in the same complex (Fig. 2C, Supplementary Fig. S3E), which was confirmed by immunofluorescence (IF) analysis (Fig. 2D).

**Fig. 2:**
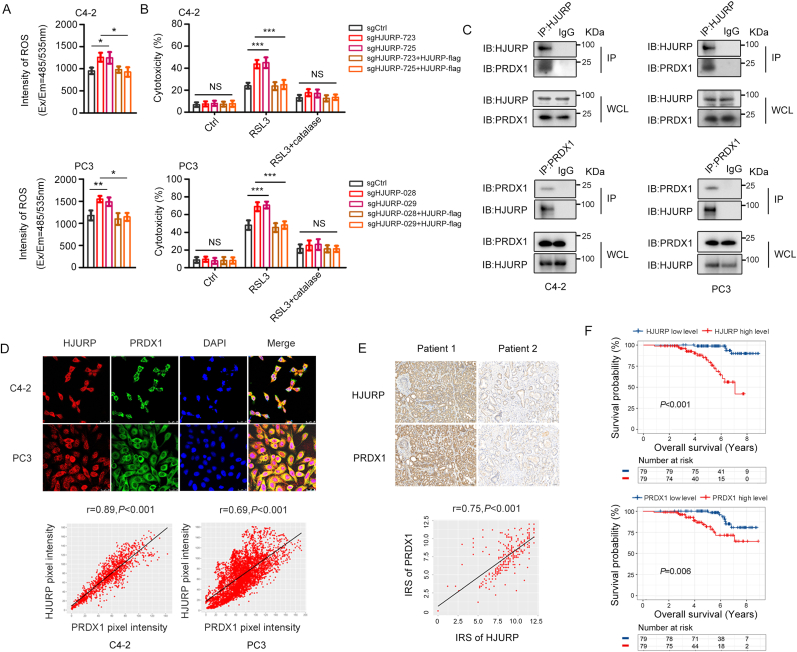
**HJURP suppressed ROS production and resided in the same complex with PRDX1, and high expression of HJURP/PRDX1 predicted poor OS in PCa patients. A.** HJURP inhibited intracellular ROS production in C4-2 and PC3 cells. **B.** HJURP knockout/re-expressed C4-2 and PC3 cells treated with RSL3 (C4-2, 1 μM; PC3, 0.5 μM) in the absence or presence of catalase (1 mg/mL) for 24 h. **C.** Co-IP assay of HJURP and PRDX1 in C4-2 and PC3 cells. **D.** IF co-staining of HJURP and PRDX1 in C4-2 and PC3 cells (scale bar: 25 μm; magnification: × 63). **E.** Representative image showing IHC staining of HJURP and PRDX1 in PCa tissues (n = 263, scale bar: 50 μm; magnification: × 200). **F.** Kaplan–Meier plots showing OS time stratified by HJURP or PRDX1 protein levels in PCa patients as determined using a tissue array analysis (n = 158). In **A** and **B**, 1-way ANOVA test was used to determine significance. In **D** and **E**, Pearson correlation analysis was used to assess the relationship between variables. Error bars indicate the SD from three independent experiments. ∗*P* < 0.05, ∗∗*P* < 0.01, ∗∗∗*P* < 0.001. IRS, Immunoreactive Score.

Moreover, the protein expression levels of HJURP and PRDX1 were positively correlated in PCa tissues (r = 0.75, *P* < 0.01, Fig. 2E). In addition, both HJURP and PRDX1 protein levels were negatively correlated with OS rates in PCa patients (Fig. 2F), and thus functioned as independent prognostic factors for OS (HJURP, HR = 2.75, 95 % CI = 1.07–7.04, *P* = 0.035; PRDX1, HR = 3.35, 95 % CI = 1.57–7.18, *P* = 0.002; Supplementary Table S3). These results demonstrated inextricable correlation between HJURP and PRDX1, and identified their roles in predicting poor outcomes in PCa patients.

Despite the inextricable correlation between HJURP and PRDX1, it remained unclear whether HJURP inhibits ROS production or sensitivity to ferroptosis inducers via PRDX1. To address this possibility, we rescued HJURP expression in HJURP-knockout C4-2 and PC3 cells while simultaneously interfering with PRDX1 expression (Fig. 3A, Supplementary Fig. S3F). Interestingly, PRDX1 knockdown significantly reversed the inhibitory effects exerted by HJURP on ROS production and sensitivity to RSL3 (Fig. 3B–F, Supplementary Figs. S3G–K). In addition, HJURP was able to inhibit H_2_O_2_-induced cell death, which could be reversed by PRDX1 knockdown (Supplementary Figs. S3L–M), further confirming that HJURP regulates ROS production via PRDX1.

**Fig. 3:**
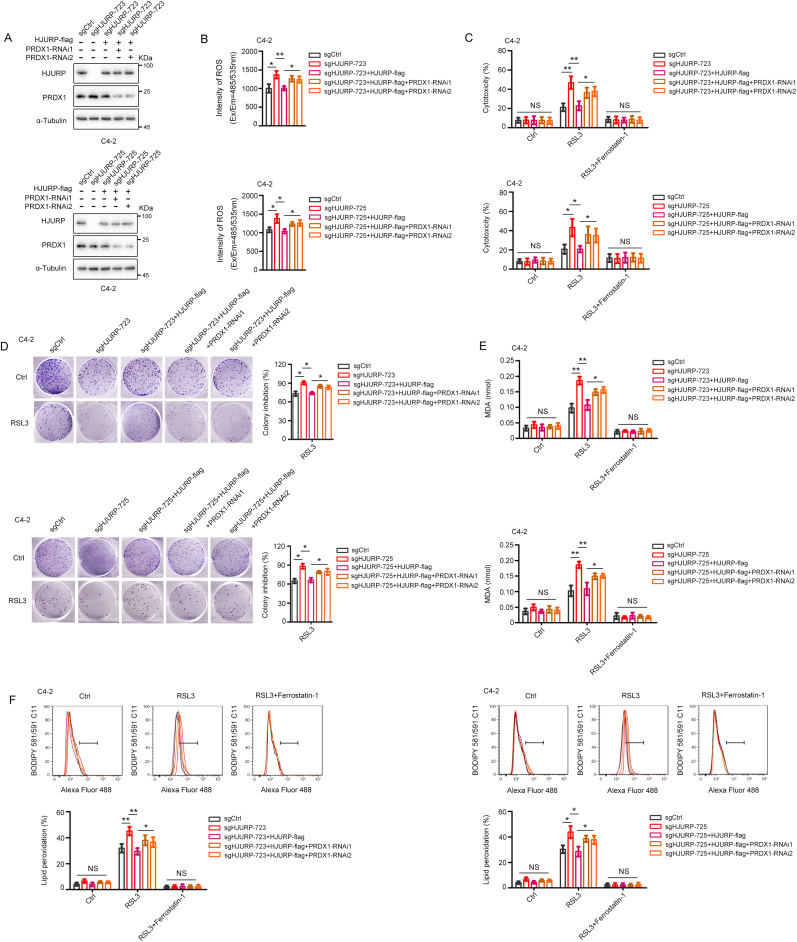
**HJURP inhibited ROS production and sensitivity to ferroptosis inducers by a PRDX1-dependent pathway. A.** Western blotting confirmed the expression of HJURP and PRDX1 in HJURP knockout C4-2 cells with/without PRDX1-RNAi. **B.** PRDX1 expression interference reversed the inhibitory effect of HJURP on ROS production. **C–F.** Cytotoxicity assay (RSL3, 1 μM, 24 h; ferrostatin-1, 2 μM, 24 h; **C**), clonogenic survival assays (RSL3, 250 nM, 12 days; **D**), MDA production (RSL3, 1 μM, 2 h; ferrostatin-1, 2 μM, 24 h; **E**), and lipid peroxidation (RSL3, 1 μM, 2 h; ferrostatin-1, 2 μM, 24 h; **F**) assays showed that the suppression of ferroptosis induced by HJURP in C4-2 cells treated with RSL3 was reversed by inhibiting PRDX1 expression. Unpaired 2-tailed *t*-test was used to analyze the differences between sgCtrl and sgHJURP or sgHJURP and sgHJURP + HJURP-flag respectively. In the rest of the figure, 1-way ANOVA test was used to determine significance. In **D**, the colony number of the RSL3 group in each HJURP-regulated group (sgCtrl. sgHJURP, etc.) was normalized to the colony number of its own Ctrl group. Colony inhibition(%) = 1-[colony number (RSL3)/colony number (Ctrl)] × 100 %. Error bars indicate the SD from three independent experiments (Error bars for clonogenic survival assays were calculated from two independent experiments). ∗*P* < 0.05, ∗∗*P* < 0.01, ∗∗∗*P* < 0.001.

Taken together, HJURP suppresses ROS production and sensitivity to ferroptosis inducers via PRDX1 in PCa cells. Moreover, higher HJURP or PRDX1 expression predicts shorter OS, and both genes serve as independent prognostic factors for OS in PCa patients.

### 2.3 HJURP forms disulfide-linked intermediates with PRDX1

To investigate how HJURP regulates PRDX1 in PCa cells, we examined the protein levels, phosphorylation, acetylation, and ubiquitination rates of PRDX1 after HJURP knockout. Surprisingly, no changes were observed in these PRDX1 post-translational modification rates (Supplementary Figs. S4A–D), despite their known role in modulating PRDX1 activity [[22], [23], [24]]. PRDX1 is a typical 2-Cys thiol-dependent peroxidase exhibiting a reductive state (sulfenic acid, PRDX1-SH), oxidized state (sulfinic acid, PRDX1-SOH), and hyperoxidized state (sulfonic acid, PRDX1-SO_2/3_H) [25]. PRDX1-SH receives peroxide to form PRDX1-SOH, and PRDX1-SOH can form disulfide-linked intermediates (PRDX1-S-S-X) with partner proteins or be overoxidized to form PRDX1-SO_2/3_H [25]. Interestingly, the disulfide-linked intermediates of typical 2-Cys PRDXs may be reduced by the Trx-reductase system, restoring their antioxidant activity [26]. However, this system is inactivated when PRDXs are overoxidized and thus in the PRDX1-SO_2/3_H form [26]. Given our findings that the HJURP levels were increased and that this protein resides in the same complex with PRDX1 in PCa cells, we hypothesized that the increase in the number of HJURP proteins leads to more disulfide-linked intermediates formed with PRDX1, attenuating PRDX1 hyperoxidation, promoting PRDX1 recycling, and ultimately enhancing PRDX1 peroxidase activity.

To test this hypothesis, we first identified disulfide-linked intermediates of HJURP (HJURP-S-S-X), which had not been confirmed by previous studies, by performing non-reducing immunoblotting. The results showed that treatment of C4-2 and PC3 cells with increasing concentrations of H_2_O_2_ induced the formation of HJURP-S-S-X, as indicated by non-reducing immunoblotting, but these intermediates disappeared in reducing immunoblots (Fig. 4A, Supplementary Fig. S4E). Notably, PRDX1 exhibited a comparable oxidation pattern (Fig. 4A, Supplementary Fig. S4E), indicating that PRDX1 oxidation may mediate the formation of HJURP-S-S-X intermediates because HJURP, which is not a specialized peroxidase, seemed unlikely to be oxidized as robustly as PRDX1, which is highly sensitive to the effects of H_2_O_2_. Moreover, the number of disulfide-linked intermediates of both PRDX1 and HJURP decreased, but the number of their monomers increased when H_2_O_2_ concentrations ≥100 μM. The possible reason is that PRDX1 is hyperoxidized at these concentrations, rendering PRDX1 inactive and unable to form disulfide bonds with HJURP. In addition, the HJURP-S-S-X levels were markedly decreased when PRDX1 expression was knocked down (Fig. 4B, Supplementary Fig. S4F). Furthermore, overexpression of the redox-inactive form of PRDX1, which carried C52S and C173S double mutations that lose the ability to form disulfide-linked intermediates [26], efficiently suppressed the formation of HJURP-S-S-X intermediates (Fig. 4C, Supplementary Fig. S4G). We then hypothesized that catalytically inactive PRDX1 may compete with active PRDX1 for binding to HJURP and thus inhibit the transfer of oxidizing equivalents. Given all that, HJURP may form disulfide-linked conjugates with PRDX1.

**Fig. 4:**
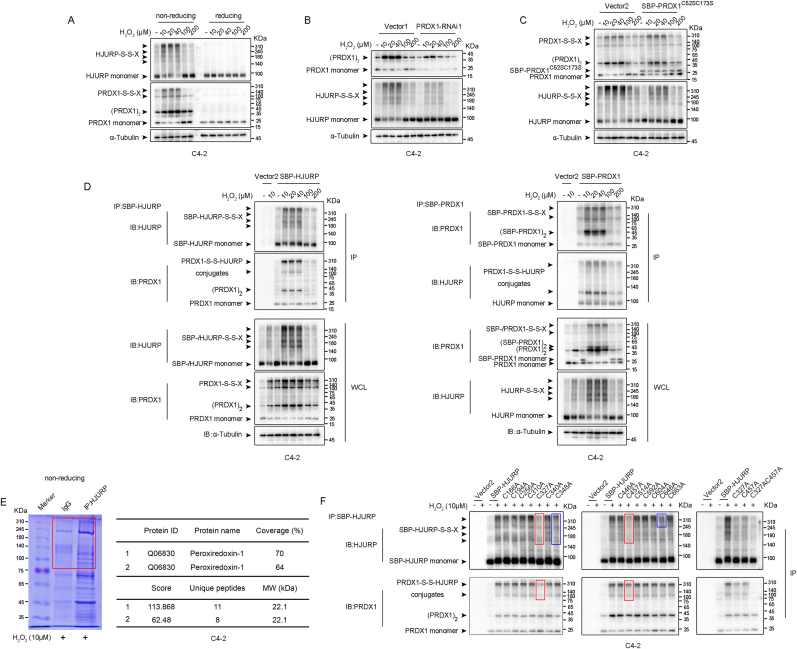
**HJURP formed disulfide-linked intermediates with PRDX1 by Cys**^**327**^**and Cys**^**457**^**. A.** Non-reducing and reducing immunoblotting of HJURP and PRDX1 in C4-2 cells treated with the indicated concentrations of H_2_O_2_ for 2 min. **B–C.** PRDX1 expression interference (**B**) or Cys^52^ and Cys^173^ mutation in PRDX1 (**C**) inhibited the formation of HJURP-S-S-X in C4-2 cells. **D.** SBP-HJURP and SBP-PRDX1 were transfected into C4-2 cells followed by treatment with the indicated H_2_O_2_ concentration for 2 min, and then, the proteins were purified using SA-based affinity purification. These purified proteins were next subjected to non-reducing immunoblotting. **E.** LC-MS/MS analysis of endogenous HJURP-S-S-X with molecular weight >75 KDa in C4-2 cells treated with 10 μM H_2_O_2_ for 2 min. **F.** SA-based affinity purification of SBP-HJURP with cysteine mutations transfected into C4-2 cells followed by treatment with 10 μM H_2_O_2_ for 2 min.

To provide direct evidence for PRDX1–HJURP disulfide binding, we tagged HJURP and PRDX1 with streptavidin (SA)-binding peptide (SBP) and expressed them in C4-2 and PC3 cells, respectively. SA-based affinity purification confirmed the formation of disulfide-linked conjugates between HJURP and PRDX1 (HJURP-S-S-PRDX1) in PCa cells (Fig. 4D, Supplementary Fig. S4H). Furthermore, we purified endogenous HJURP from C4-2 cells treated with 10 μM H_2_O_2_ for 2 min and subjected the HJURP-S-S-X, identified with a molecular weight >75 KDa, to LC-MS/MS analysis. Consistent with the results presented above, many unique PRDX1 peptides were evident in the HJURP disulfide-linked conjugates (Fig. 4E), providing further confirmation of the formation of disulfide-linked intermediates between HJURP and PRDX1 in PCa cells.

Next, we identified the cysteines in HJURP that are involved in the formation of HJURP-S-S-PRDX1 intermediates. HJURP carries 14 cysteines, and each of these residues was individually mutated. The results showed that mutation of Cys^327^, Cys^348^, and Cys^457^ profoundly diminished the HJURP-S-S-X levels, and the Cys^646^ mutation reduced the levels of only the HJURP-S-S-X peptide with a mass >310 KDa (Fig. 4F). However, only Cys^327^ and Cys^457^ mutations inhibited the formation of PRDX1-S-S-HJURP conjugates (Fig. 4F). Considering these results, we introduced mutations into both Cys^327^ and Cys^457^, which completely abolished the formation of PRDX1-S-S-HJURP conjugates (Fig. 4F).

In summary, HJURP forms disulfide-linked intermediates with PRDX1 in PCa cells, and Cys^327^ and Cys^457^ in HJURP are the cysteine residues that form disulfide bonds with PRDX1.

### 2.4 HJURP inhibits sensitivity to ferroptosis inducers by enhancing the peroxidase activity of PRDX1 via disulfide binding

Given the presence of HJURP-S-S-PRDX1 intermediates in PCa cells, we next investigated whether HJURP by forming disulfide bonds with PRDX1 affects the peroxidase activity of PRDX1. Notably, the dimer intermediate is positively correlated with PRDX1 peroxidase activity, while PRDX1-SO_3_H is negatively correlated with this activity [27,28]. Therefore, enhanced PRDX1 peroxidase activity manifests as an increase in the number of PRDX1 dimer intermediates and a decrease in PRDX1-SO_3_H levels.

According to the results of the Co-IP assay and non-reducing immunoblotting, PRDX1-SO_3_H levels were increased, while the number of PRDX1 dimer intermediates was decreased after HJURP knockout in C4-2 and PC3 cells (Fig. 5A and B, Supplementary Figs. S5A–B). These outcomes were reversed when HJURP expression was rescued (Fig. 5C and D, Supplementary Figs. S5C–D). This result suggested that HJURP enhances PRDX1 peroxidase activity. Notably, rescuing HJURP^C327AC457A^ expression in HJURP-knockout cells did not reverse the changes to the number of PRDX1 dimer intermediates or PRDX1-SO_3_H levels; instead, it led to an increase in the number of PRDX1-SO_3_H molecules and a decrease in the number of PRDX1 dimer intermediates (Fig. 5E and F, Supplementary Figs. S5E–F). These findings indicate that HJURP^C327AC457A^, which loses the ability to form disulfide bonds with PRDX1, competes with PRDX1 partner proteins for PRDX1 binding, suppressing disulfide bond formation. Consequently, this interaction inhibits the redox cycling of PRDX1, promotes its hyperoxidation, and ultimately weakens its antioxidant activity. In addition, HJURP, but not HJURP^C327AC457A^, promoted the formation of PRDX1-S-S-Trx1, suggesting that more disulfide intermediates of PRDX1 are reduced by the Trx system and ultimately reconverted to PRDX1-SH [29] (Supplementary Figs. S6A–B). Collectively, these results show that HJURP enhances the peroxidase activity of PRDX1 by forming disulfide bonds with it.

**Fig. 5:**
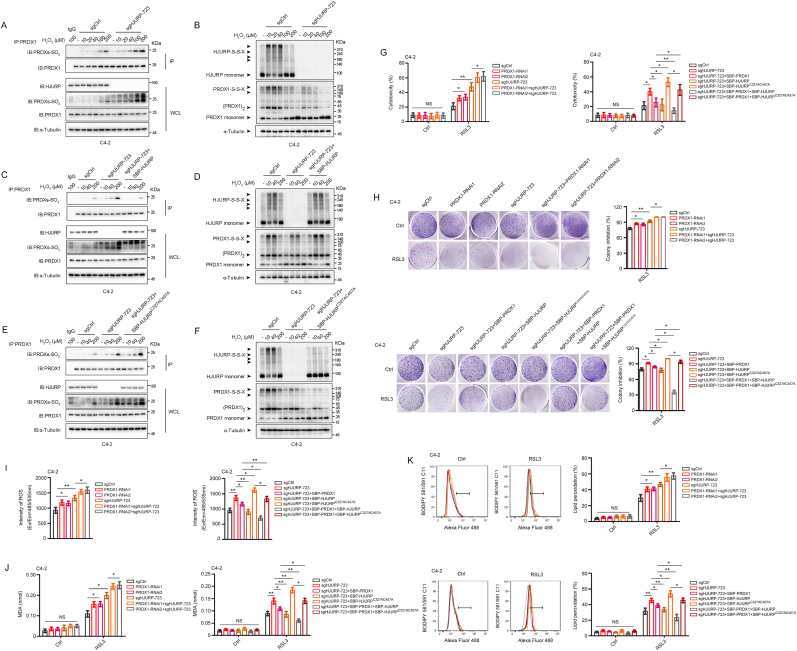
**HJURP cooperated with PRDX1 by disulfide binding to enhance PRDX1 peroxidase activity and inhibit the sensitivity to ferroptosis inducers in PCa cells. A-D.** Knockout of HJURP promoted PRDX1 hyperoxidation (**A**) while suppressing the formation of PRDX1 disulfide-linked conjugates **(B)** in C4-2 cells; however, rescuing HJURP expression reversed that (**C-D**). **E-F.** HJURP^C327AC457A^ led to increased PRDX1 hyperoxidation (**E**) but decreased levels of PRDX1 disulfide-linked conjugates (**F**). **G-K.** Cytotoxicity (RSL3, 1 μM, 24 h; **G**), clonogenic survival assays (RSL3, 250 nM, 12 days; **H**), DCFDA (RSL3, 1 μM, 24 h; **I**), MDA (RSL3, 1 μM, 24 h; **J**), and lipid peroxidation (RSL3, 1 μM, 24 h; **K**) assays showed that HJURP, but not HJURP^C327AC457A^, synergized with PRDX1 to suppress cell death induced by RSL3, scavenge intracellular ROS or inhibit lipid peroxidation in C4-2 cells treated with RSL3. 1-way ANOVA test was used to analyze the differences between sgCtrl and PRDX1-RNAi or sgHJURP and PRDX1-RNAi + sgHJURP, respectively. In the rest of the figure, unpaired 2-tailed *t*-test was used to determine significance. In H, the colony number of the RSL3 group in each HJURP-regulated group (sgCtrl. sgHJURP-723, PRDX1-RNAi, etc.) was normalized to the colony number of its own Ctrl group. Colony inhibition(%) = 1-[colony number (RSL3)/colony number (Ctrl)] × 100 %. Error bars indicate the SD from three independent experiments (Error bars for clonogenic survival assays were calculated from two independent experiments). ∗*P* < 0.05, ∗∗*P* < 0.01, ∗∗∗*P* < 0.001.

To confirm this conclusion, we analyzed the changes in ROS production and sensitivity to ferroptosis inducers in C4-2 and PC3 cells after co-expression or co-depletion of HJURP and PRDX1(Supplementary Figs. S6C–D). Simultaneous depletion of both HJURP and PRDX1 significantly increased ROS production and enhanced the sensitivity of PCa cells to RSL3 compared to the outcomes after the depletion of either HJURP or PRDX1 alone. Conversely, co-expression of both proteins inhibited ROS production and the sensitivity of PCa cells to RSL3 (Fig. 5G–K, Supplementary Figs. S7 and S8). Notably, HJURP^C327AC457A^ did not enhance PRDX1 peroxidase activity of PRDX1, instead, it significantly suppressed it (Fig. 5G–K, Supplementary Figs. S7 and S8), suggesting that HJURP enhances PRDX1 peroxidase activity only in the presence of HJURP-S-S-PRDX1 intermediates.

Furthermore, we evaluated the effects of altering HJURP and PRDX1 levels on the sensitivity of PCa cells to ferroptosis inducers *in vivo*. Depletion of both HJURP and PRDX1 led to a greater inhibitory effect on C4-2 cell growth induced by PACMA31 (71.0 % inhibition of xenograft tumor weight and 76.4 % inhibition of xenograft tumor volume), a stable GPX4 inhibitor *in vivo* [30], compared to the effects of the depletion of either HJURP (51.0 % inhibition of xenograft tumor weight and 61.0 % inhibition of xenograft tumor volume) or PRDX1 (54.2 % inhibition of xenograft tumor weight and 62.5 % inhibition of xenograft tumor volume) alone (Fig. 6A–D). Moreover, immunohistochemical (IHC) staining of xenograft tissue demonstrated that the levels of 4-HNE, the product of lipid peroxidation, were the highest in the group in which both HJURP and PRDX1 had been depleted (Fig. 6E).

**Fig. 6:**
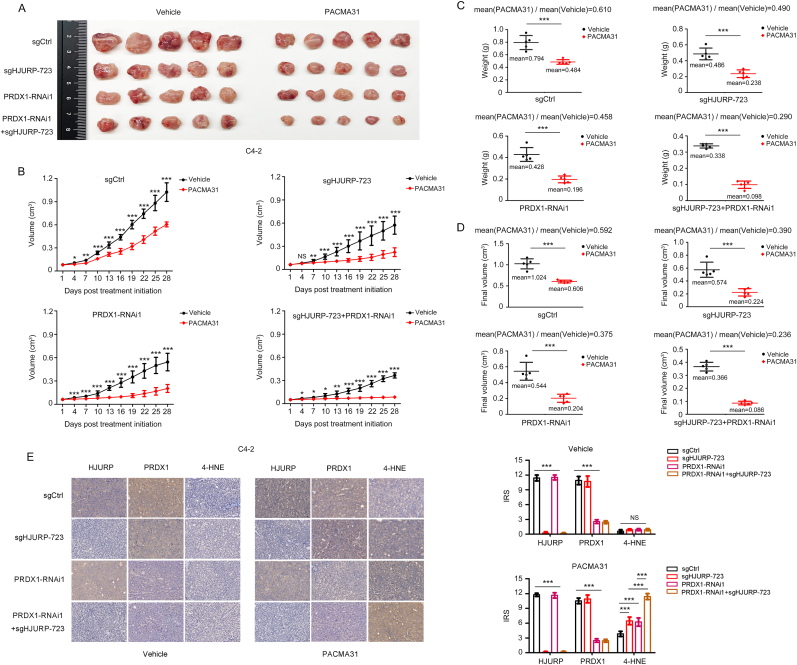
**Inhibition of HJURP and PRDX1 significantly enhanced the anti-tumor activity of PACMA31 *in vivo*. A-B.***In vivo* growth of subcutaneous C4-2 tumors treated i.p. with PACMA31 (10 mg/kg, twice/week) compared with vehicle groups. **C-D.** Ratio of mean xenograft tumor weight **(C)** or final volume **(D)** in PACMA31 group to mean xenograft tumor weight **(C)** or final volume **(D)** in vehicle group. **E.** Representative IHC staining of HJURP, PRDX1, and 4-HNE in C4-2 cell xenograft tissues (scale bar: 50 μm; magnification: × 200). The IRS scores of HJURP, PRDX1 and 4-HNE in the vehicle group and HJURP, PRDX1 in the PACMA31 treatment group were analyzed by 1-way ANOVA test. In the rest of the figure, unpaired 2-tailed *t*-test was used to determine significance. Error bars indicate the SD of 5 xenograft tumors per group. ∗*P* < 0.05, ∗∗*P* < 0.01, ∗∗∗*P* < 0.001. IRS, Immunoreactive Score.

Taken together, HJURP forms disulfide bonds with PRDX1 and enhances its peroxidase activity, leading to the inhibition of ROS production and ultimately conferring resistance to ferroptosis inducers in PCa cells (Fig. 7).

**Fig. 7:**
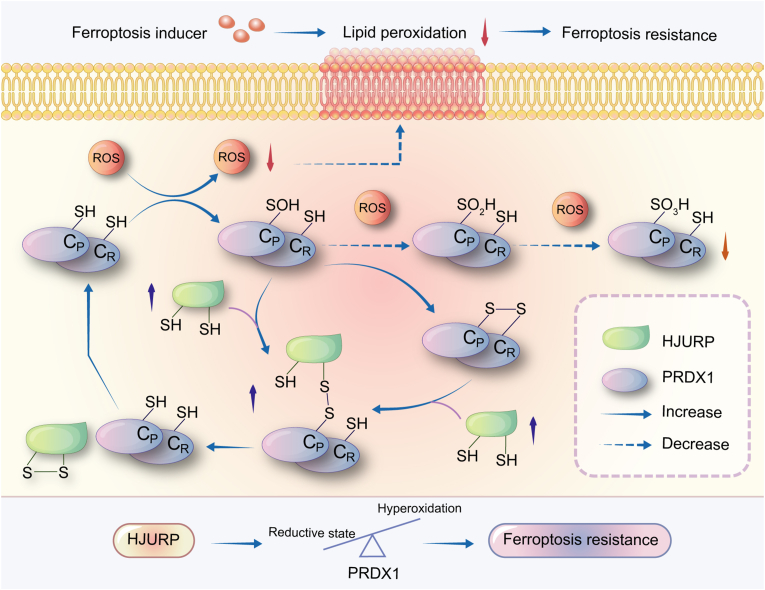
**HJURP inhibited PCa cells sensitivity to ferroptosis inducers via the PRDX1/ROS pathway.** Increased HJURP levels resulted in increased formation of HJURP-S-S-PRDX1 by direct disulfide binding with PRDX1-SOH or by disulfide bond exchange with PRDX1-S-S-PRDX1, which promoted PRDX1 redox cycling and caused less PRDX1 hyperoxidation. Disulfide binding enhanced the peroxidase activity of PRDX1, leading to a decrease in ROS levels, and ultimately inhibiting the sensitivity of PCa cells to ferroptosis.

## 3 Discussion

Oxidative stress, iron overload, and lipid metabolism are key regulatory factors in ferroptosis [[31], [32], [33]]. Compared to those in normal cells, iron and ROS levels are abnormally increased in PCa cells because of their high metabolic rate [34]. Furthermore, upregulation of lipid metabolism genes has been found in both early and advanced PCa, indicating that lipid metabolism is the main source of energy production in PCa cells [35,36]. Therefore, targeting ferroptosis may be a viable strategy for treating PCa. Indeed, increasing evidence has shown that inducing ferroptosis inhibits the growth of PCa cells, and specific ferroptosis inducers such as Erastin, RSL3, and sulfasalazine have been confirmed to induce the death of PCa cells [11,12]. Moreover, combining abiraterone or enzalutamide with RSL3 or Erastin has shown synergistic ferroptosis-inducing effects on PCa cells [37], highlighting ferroptosis as a promising therapeutic strategy for patients with PCa. Identifying patients who are sensitive to ferroptosis may facilitate the use of PCa precision therapy.

In this study, we demonstrated that HJURP cooperates with PRDX1 to detoxify cells by reducing ROS production and induces resistance to ferroptosis inducers in PCa cells, suggesting that HJURP/PRDX1 may be used as markers to measure the ferroptosis sensitivity of PCa patients. Mechanistically, HJURP forms disulfide-linked intermediates with PRDX1, inhibiting PRDX1 hyperoxidation and promoting redox cycling, thereby enhancing PRDX1 peroxidase activity.

PRDX1 is a typical 2-Cys PRDX with Cys^52^P-SH-peroxidizing and Cys^173^R–SH–resolving activity [25]. Cys^52^P-SH contributes to the oxidative modification of interaction partners in several ways [26]: 1) PRDX1-Cys^52^P–SOH establishes a disulfide bond with Cys^173^R–SH in another PRDX1 protein, leading to the formation of homodimers. These homodimers subsequently exchange disulfide bonds with partner proteins. 2) PRDX1-Cys^52^P–SOH directly establishes disulfide bonds with active cysteines in interacting proteins, resulting in the formation of heterodimers. 3) PRDX1-Cys^52^P–SOH is peroxidized to yield PRDX1-Cys^52^P–SO_2_H or PRDX1-Cys^52^P–SO_3_H. Both PRDX1 homodimers and heterodimers can be reduced by Trx, Trx-reductase, and NADPH, restoring PRDX1 antioxidant activity [28]. However, PRDX1-Cys^52^P–SO_2_H and PRDX1-Cys^52^P–SO_3_H, which are the inactivated states of PRDX1, cannot be reduced by Trx [26]. Notably, PRDX1-Cys^52^P–SO_2_H can contribute to a reduction reaction induced by ATP, Mg^2+^, and Srx, although it is slower and less efficient [38]. On the other hand, PRDX1-Cys^52^P–SO_3_H binding is irreversible [26]. According to our previous study, the HJURP levels is increased in PCa cells [16]. Therefore, we believe that the increased amount of HJURP competitively interacts with PRDX1-Cys^52^P–SOH or PRDX1 homodimers, forming a disulfide-linked intermediate, ablating the hyperoxidation of PRDX1 because of the higher proportion of PRDX1-Cys^52^P–SOH constructs disulfide heterodimer with HJURP. Moreover, once PRDX1-Cys^52^P–SOH has established a disulfide structure, it becomes less susceptible to hyperoxidation [39]. This potential mechanism may explain how HJURP protects PRDX1 from hyperoxidation. Notably, PRDX1 is more susceptible to hyperoxidation, which not only occurs in the presence of nonphysiologically high levels of H_2_O_2_ but also in the presence of H_2_O_2_ concentrations are <1 μM or even in the absence of H_2_O_2_ [39], indicating that PRDX1 hyperoxidation can take place under normal cell culture conditions. Therefore, the upregulated expression of HJURP is essential for the antioxidant defense of PCa cells as it inhibits PRDX1 peroxidation.

HJURP is known to be involved in DNA double-strand break repair, centromere formation, and tumorigenesis [[13], [14], [15], [16],^40^]. In this study, we demonstrated a novel role for HJURP in inducing resistance to ferroptosis inducers via the PRDX1/ROS pathway. Interestingly, the regulation of ROS production by HJURP varies in different kinds of cancer: HJURP inhibits ROS production via the PPARγ-SIRT1 feedback loop in bladder cancer [41], while it enhances ROS stress in renal cell carcinoma [42]. In our study, HJURP scavenged ROS by cooperating with PRDX1. The heterogeneity of different tumors may be the reason for these differences. Moreover, the differences of HJURP between various tumors not only present in ROS regulation but also in subcellular localization. In glioma [43], HJURP has been detected in both the cytoplasm and nucleus. In colorectal cancer [44] and pancreatic cancer [15], it localized to the nucleus. In PCa, however, we confirmed that HJURP is located mainly in the cytoplasm [16]. These findings suggest that HJURP exhibits different functions depending on the tumor type, likely due to its diverse subcellular localization.

In summary, our study reveals the role of HJURP in ferroptotic resistance by synergizing with PRDX1 to scavenge ROS in PCa cells. Thus, HJURP/PRDX1 may be used as biomarkers for predicting the response of PCa cells to ferroptosis inducers and are promising therapeutic targets for overcoming PCa ferroptotic resistance. However, it is worth noting that whether HJURP also influences the activity of other key antioxidant enzymes, such as glutathione s-transferases, glutathione peroxidases, and protein disulfide isomerases, remains an open question that warrants further comprehensive and systematic investigation.

## 4 Materials and methods

### 4.1 Cell cultures

C4-2 and PC3 cells were obtained from the American Type Culture Collection (Manassas, USA). Both cell lines were cultured in RPMI-1640 medium (HyClone, USA) supplemented with 10 % fetal bovine serum (FBS, Bovogen, Australia). The cells were maintained in a humidified incubator at 37 °C with 5 % CO_2_. Prior to experimentation, the cells were authenticated by short tandem repeat profiling and evaluated to ensure that they were free from mycoplasma contamination.

### 4.2 CRISPR-Cas9 assay

Lentiviruses carrying HJURP single guide RNA (sgRNA) and control sgRNA were purchased from GenePharma Biotech Company (Shanghai, China). HJURP-knockout C4-2 and PC3 cells were selected after puromycin treatment (5 μg/ml) for 15 days, and single cell-derived HJURP-knockout clones were generated by CloneSelect Single-Cell Printer (UP. SIGHT, CYTENA, USA). The knockout results were confirmed by western blotting and Sanger sequencing. The target sequences of sgHJURP and sgCtrl are presented in Supplementary Table S1.

### 4.3 Lentivirus and plasmids

Lentivirus: Lentiviruses for PRDX1 knockdown and control were purchased from GenePharma Biotech Company (Shanghai, China). PRDX1 knockdown and control lentiviruses were transfected into cells based on the manufacturer's instructions. Stably transfected cells were selected after puromycin treatment at a concentration of 5 μg/ml for 15 days.

Plasmids: HJURP and PRDX1 cDNAs were amplified by PCR and cloned into a PCDNA-3.1-Flag or SBP-PCDNA-3.1 vector, with an empty vector used as the control. Transfections were carried out using Lipofectamine 3000 (Invitrogen, USA) according to the manufacturer's protocol. Specific point mutations in HJURP and PRDX1 were introduced with a QuikChange Site-Directed Mutagenesis Kit (Agilent, USA).

Detailed information on the lentivirus and plasmids is provided in Supplementary Table S1.

### 4.4 Cell death assay

The activity of lactate dehydrogenase (LDH) in the incubation medium was analyzed using the LDH-Cytotoxicity Assay Kit II (Abcam, ab65393, USA). According to the protocol, C4-2 and PC3 cells were seeded into 96-well plates (2 × 10^4^ cells/well) with the indicated treatment. Next, the incubation medium (10 μl/well) was transferred into an optically clear 96-well plate with 100 μl of LDH Reaction Mix and incubated for 30 min at room temperature. The absorbance was read at 450 nm using a microplate reader (Bio-Rad, USA).

Cytotoxicity (%) = (Test Sample – Low Control)/(High Control – Low Control) × 100. Low Control, the incubation medium (10 μl) from wells (2 × 10^4^ cells/well) without any treatment. High Control, the incubation medium (10 μl) from wells (2 × 10^4^ cells/well) with 10 μl Lysis Buffer II/Cell Lysis Solution.

### 4.5 Cell viability assay

Cell viability was assessed using a Cell Counting Kit-8 (CCK8) assay (Dojindo, Japan) following a previously described method [16]. In brief, C4-2 and PC3 cells were seeded into 96-well plates (2 × 10^3^ cells/well) for the indicated times, and the medium was replaced with fresh culture medium containing 10 % CCK8 solution. The absorbance was read at 450 nm using a microplate reader (Bio-Rad, USA).

### 4.6 Colony formation assay

PCa cells were seeded into six-well plates at a density of 2 × 10^3^ cells per well. After 12 days of exposure to the specified treatment, the cells were washed three times with phosphate-buffered saline (PBS) and fixed with 4 % paraformaldehyde solution for 15 min. After fixation, the cells were stained with 0.5 % crystal violet solution (KeyGEN, China) for 10 min. Finally, the plates were then rinsed with deionised water in preparation for subsequent analysis.

### 4.7 Reducing and non-reducing western blotting

Reducing western blotting was performed following a previously described protocol [16]. For non-reducing western blotting, PCa cells were cultured in 6-well plates and treated with different concentrations of H_2_O_2_ for 2 min. Next, the H_2_O_2_ was removed and the action of free thiols was blocked by treating the cells with 20 mM methyl methanethiosulfonate (MMTS) in 1 × PBS for 10 min. After blocking, the cells were washed 3 times with 1 × PBS and lysed using lysis buffer containing 0.1 % protease inhibitor, 1 % phosphatase inhibitor, and 1 % phenylmethanesulfonyl fluoride. The supernatants were mixed with 4 × loading buffer without mercaptoethanol or other reducing agents and heated to 95 °C for 10 min. The samples were then subjected to SDS-PAGE and transferred to polyvinylidene difluoride membranes (Merck Millipore, USA). The membranes were probed with primary antibody overnight at 4 °C, followed by incubation with appropriate secondary antibody. Visualization was performed using a ChemiDoc Imaging System (Bio-Rad, USA).

Detailed information about the antibodies and reagents used in this study can be found in Supplementary Table S1.

### 4.8 Wound healing assay

C4-2 and PC3 cells were seeded into 6-well plates (6 × 10^5^ cells/well) and allowed to reach confluence. A scratch was made in each well with a pipette tip. The scratches were imaged at 0 h and 48 h using an inverted phase-contrast microscope (Olympus Optical, Japan). Wound width (%) = wound width at 48 h/wound width at 0 h.

### 4.9 Migration and invasion assays

A total of 5 × 10^4^ cells were plated into the upper chamber and the polycarbonate filters were coated with (invasion assay) or without (migration assay) Matrigel (BioCoat, USA). The lower chamber was supplemented with fresh medium containing 10 % FBS. The cells were cultured for 48 h, and methanol and crystal violet were used to fix and stain the cells that migrated to the lower well, respectively. The stained cells were photographed for future counting and analysis.

### 4.10 Lipid peroxidation assay

C4-2 and PC3 cells were seeded into 6-well plates (3 × 10^5^ cells/well) and cultured with 1 ml of fresh medium containing BODIPY 581/591 C11 (5 μM, D3861, Thermo Fisher, USA) for 30 min at 37 °C with 5 % CO_2_. The cells were then trypsinized, washed, and resuspended in 500 μl of 1 × PBS and used for flow cytometry analysis (Beckman CytoFLEX, USA). At least 1 × 10^4^ cells per condition were analyzed.

### 4.11 Malondialdehyde (MDA) assay

The levels of MDA in C4-2 and PC3 cells were measured with a Lipid Peroxidation (MDA) Assay Kit (Abcam, ab118970). Based on the manufacturer's instructions, 2 × 10^6^ cells were lysed in MDA lysis buffer, and the supernatants of each sample were allowed to react with thiobarbituric acid (TBA) to generate an MDA-TBA adduct. A microplate reader was used to measure the absorbance of MDA-TBA at 532 nm.

### 4.12 Iron and GSH assays

Iron assay: Iron Assay Kit (Abcam, ab83366) was applied to analyze the Fe^2+^ concentration of C4-2 and PC3 cells. According to the manufacturer's instructions, cells (1 × 10^6^) were resuspended in 100 μl of iron assay buffer and lysed by sonication. After centrifugation, the supernatants were incubated with the iron probe for 30 min at 37 °C. The absorbance of each sample was measured at 593 nm.

GSH assay: GSH/GSSG Ratio Detection Assay Kit II (Abcam, ab205811) was used to determine the GSH content in C4-2 and PC3 cells. Based on the manufacturer's protocol, cells (5 × 10^4^) were lysed in 1 × PBS containing 0.5 % NP-40, and the resulting supernatants were incubated with the GSH assay mixture (GAM) solution for use in GSH analysis at room temperature for 60 min. The samples were analyzed with a fluorometric microplate reader (Bio-Rad, USA) at Ex/Em = 490/520 nm, and the GSH amounts were calculated based on the standard curve.

### 4.13 Evaluation of ROS production

Intracellular ROS production was analyzed using a 2′,7′-dichlorodihydrofluorescein diacetate (DCFDA/H_2_DCFDA)-Cellular ROS Assay Kit (Abcam, ab113851). Cells were seeded into 96-well plates (2.5 × 10^4^ cells/well) and incubated at 37 °C with 5 % CO_2_. The next day, the medium was replaced with DCFDA solution (40 μM) and incubated at 37 °C for 45 min. After removing the DCFDA solution, the cells were washed 3 times with 1 × PBS, and then, 1 × buffer was added to each well. The 96-well plates were analyzed with a fluorometric microplate reader (Bio-Rad, USA) at Ex/Em = 485/535 nm.

### 4.14 Co-IP and SA-based affinity enrichment assays

Co-IP: This assay was performed following a previously described protocol [16]. Briefly, cells were lysed and centrifuged, and the resulting supernatants were used for immunoprecipitation with primary antibody and incubated in a shaking incubator at 4 °C for 4 h. Next, the samples were mixed with protein-A/G mix beads (Thermo Scientific) and incubated overnight at 4 °C. The following day, the beads were collected and washed 3 times with lysis buffer, and the samples were further prepared for use in western blotting.

SA-based affinity enrichment assays [17]: Plasmids with a SBP-tag were transfected into C4-2 and PC3 cells. After 2 days, the cells were treated with different concentrations of H_2_O_2_ for indicated times. Subsequently, the cells were washed 3 times with 1 × PBS at 4 °C and incubated with 20 mM MMTS for 10 min to block free thiols. Following another 3 washes with 1 × PBS, the cells were lysed in 500 μl of TBS (50 mM Tris and 150 mM NaCl, pH = 7.4) containing 1 % Triton X-100, 0.1 % protease inhibitor, 1 % phosphatase inhibitor, and 1 % phenylmethanesulfonyl fluoride at 4 °C for 30 min. After centrifugation at 16,000×*g* for 10 min at 4 °C, the supernatants were incubated with 25 μl of SA-Sepharose beads (Thermo Scientific) for 6 h. The beads were collected, washed first with TBS containing 1 % Triton X-100, 1 M urea, then with TBS containing 1 % Triton X-100, and finally with TBS containing 0.1 % Triton X-100. SBP-tagged proteins were eluted from the beads with 30 μl of biotin (4 mM) in TBS. The eluted samples were mixed with 2 × loading buffer without mercaptoethanol or other reducing agents, heated to 95 °C for 10 min, and subjected to SDS-PAGE and non-reducing immunoblotting.

### 4.15 IF staining

To determine the co-localization between intracellular HJURP and PRDX1, we performed IF staining of endogenous HJURP and PRDX1 in C4-2 and PC3 cells. Briefly, the cells were fixed with 4 % paraformaldehyde for 30 min, washed with 1 × PBS, and then permeabilized with 0.1 % Triton X-100 for 15 min at room temperature. Next, the cells were blocked with 1 % BSA for 30 min and incubated with 50 μl of primary antibody overnight at 4 °C. The following day, the cells were washed 3 times with 1 × PBS and incubated with 50 μl of secondary antibody for 1 h in the dark. After removing the secondary antibody and washing the cells 3 times with 1 × PBS, the cell nuclei were stained with DAPI. Fluorescence images were captured using a super-resolution confocal microscope (Leica TCS SP8, Germany) and analyzed by LAS AF Lite analysis software. The pixel intensity correlation between HJURP and PRDX1 was calculated with ImageJ software (ImageJ 2.9.0).

### 4.16 IHC staining

A total of 263 patients diagnosed with PCa were included in this study. The cohort comprised 105 patients from the Third Affiliated Hospital of Sun Yat-sen University (Guangzhou, China) and 158 patients from a tissue array obtained from Wuhan Servicebio Biotech Co. (Shanghai, China). The information on these patients is presented in Supplementary Table S2. Ethics approval for this study was obtained from the Ethics Review Committee of the Third Affiliated Hospital of Sun Yat-sen University (RG2023-010-01). Additionally, the study was registered in the Chinese Clinical Trial Registry (ChiCTR2300072528). All patients provided written informed consent in accordance with the guidelines of the Declaration of Helsinki. The methods used for IHC staining and immunoreactive score evaluation have been previously reported [16].

### 4.17 LC-MS/MS

LC-MS/MS (Supplementary Fig. S3D): To determine the intracellular complex of HJURP in PCa cells, C4-2 cells (1 × 10^6^ per sample) were lysed, and the supernatants were used for Co-IP with primary anti-HJURP antibody (Abcam, ab100800) or IgG (Abcam, ab6715). The Co-IP methods are described above. The samples were mixed with 2 × loading buffer containing 20 % mercaptoethanol, heated to 95 °C for 10 min, and subjected to reductive SDS-PAGE. The gel bands of all the proteins were carefully cut, removed and sent to Sangon Biotech Company (Shanghai, China) for LC-MS/MS analysis. This assay was performed in duplicate.

LC-MS/MS (Fig. 4E): To confirm that HJURP formed disulfide-linked intermediates with PRDX1, C4-2 cells (3 × 10^6^ per sample) were treated with H_2_O_2_ (10 μM) for 2 min, followed by treatment with MMTS (20 mM) for 10 min to block free thiols. The cells were lysed and centrifuged, and the resulting supernatants were incubated with primary anti-HJURP antibody (Abcam, ab100800) to pull down endogenous HJURP. IgG (Abcam, ab6715) was used as the antibody control. The Co-IP protocol is presented above. The samples were mixed with 2 × loading buffer without mercaptoethanol or other reductive agents, heated to 95 °C for 10 min, and then subjected to non-reducing SDS-PAGE. The gel containing the disulfide-linked intermediates of HJURP with molecular weights >75 KDa was analyzed by LC-MS/MS at the Instrumental Analysis & Research Center of Sun Yat-sen University (Guangzhou, China). This assay was performed in duplicate.

### 4.18 Mouse xenograft assay

All animal used in this study were 4- to 6-week-old BALB/C nude male mice obtained from the Sun Yat-sen University Laboratory Animal Center (Guangzhou, China)**.** The mice were housed in a specific-pathogen-free environment and provided with sterile food and water. Ethical approval for the animal experiments was obtained from the Institutional Animal Care and Use Committee, Sun Yat-Sen University (SYSU-IACUC-2024-001628). The mice were injected subcutaneously with C4-2 cells (5 × 10^6^) (the mice were assigned to 4 groups: sgCtrl, sgHJURP-723, PRDX1-RNAi1, and sgHJURP-723+PRDX1-RNAi1 group with 10 mice per group). When tumors were palpable (50–80 mm^3^), the mice in each group were randomly assigned to DMSO and PACMA31 (10 mg/kg, intraperitoneal injection, twice/week) treatment groups (5 mice per group). The treatment continued for 4 weeks, and tumor size was measured every 3 days. At the end of the experimental procedure, the mice were euthanized under deep anesthesia with sodium pentobarbital (40 mg/kg) (Sigma). The tumors were removed, weighed and processed for further analyses, and the carcasses were disposed of at the Laboratory Animal Center. Tumor volume = π/6 × length × width^2^.

Guided by guidelines [45], we completed photographic documentation of the excised tumor tissues within 5 min post-excision and immediately proceeded to freeze them in liquid nitrogen for temporary storage. Additionally, we opted for frozen section over the conventional paraffin-embedded section for immunohistochemical staining on the same day, to maximally preserve antigen activity and prevent *ex vivo* changes in lipid peroxidation.

### 4.19 Statistical analysis

The data are presented as the mean ± standard deviation (SD). Statistical analysis was performed using the Statistical Package for the Social Sciences (SPSS) v.25.0 software (SPSS Inc., Chicago, USA). The correlations between OS and HJURP or PRDX1 were evaluated using the log-rank test based on the Kaplan–Meier method. The median immunoreactivity score (IRS) of HJURP or PRDX1 was regarded as the cutoff point to divide the PCa patients into high- and low-expression groups. Univariate analysis was initially used to analyze the OS data, and then covariates with a *P* value ≤ 0.05 were analyzed by multivariate Cox regression. The pixel intensity correlation between HJURP and PRDX1 is presented as a Pearson product-moment correlation coefficient. For the cell death assay, cell viability assay, lipid peroxidation assay, MDA assay, wound healing assay, migration and invasion assays, GSH assay, intracellular ROS assay, and animal experiments, 1-way ANOVA or unpaired 2-tailed *t*-test was performed to assess the significance of differences. *P* < 0.05 was regarded as statistically significant. ∗ represents *P* < 0.05, ∗∗ represents *P* < 0.01, and ∗∗∗ represents *P* < 0.001.

## CRediT authorship contribution statement

**Wenjie Lai:** Writing – review & editing, Writing – original draft, Visualization, Methodology, Conceptualization. **Weian Zhu:** Writing – review & editing, Writing – original draft, Validation, Methodology. **Jianjie Wu:** Investigation, Formal analysis. **Jiongduan Huang:** Software, Data curation. **Xiaojuan Li:** Resources, Funding acquisition. **Yun Luo:** Writing – review & editing, Funding acquisition. **Yu Wang:** Resources. **Hengda Zeng:** Investigation. **Mingqiang Li:** Writing – review & editing, Supervision. **Xiaofu Qiu:** Writing – review & editing, Supervision, Project administration, Funding acquisition. **Xingqiao Wen:** Writing – review & editing, Supervision, Project administration, Funding acquisition, Conceptualization.

## Ethics approval and consent to participate

The studies involving human participants were approved by the Ethics Review Committee of the Third Affifiliated Hospital of Sun Yat-sen University (RG2023-010-01) and registered in the Chinese Clinical Trial Registry (ChiCTR2300072528). Written informed consent was received from patients according to the guidelines of the Declaration of Helsinki. All animal studies have been approved and performed in accordance with the Institutional Animal Care and Use Committee, Sun Yat-Sen University (SYSU-IACUC-2024-001628). The carcasses were disposed of by the Laboratory Animal Center.

## Declaration of competing interest

The authors declare that they have no known competing financial interests or personal relationships that could have appeared to influence the work reported in this paper.

## Figures and Tables

**figs1:**
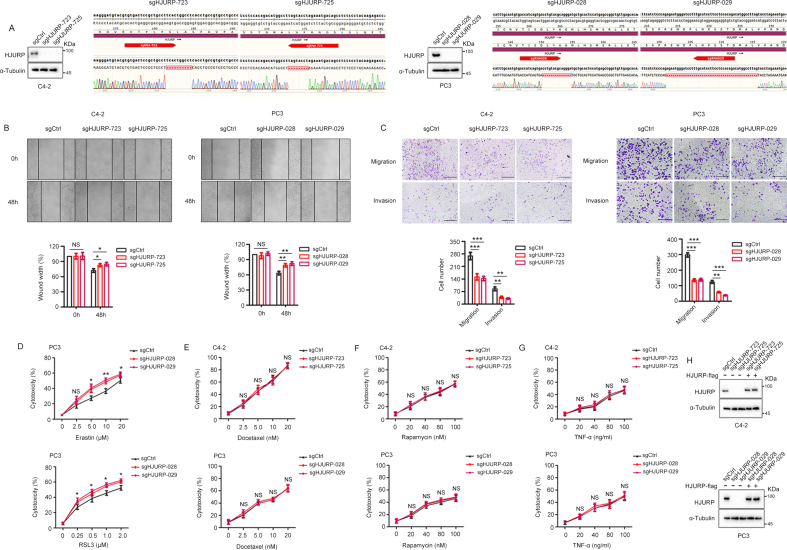


**figs2:**
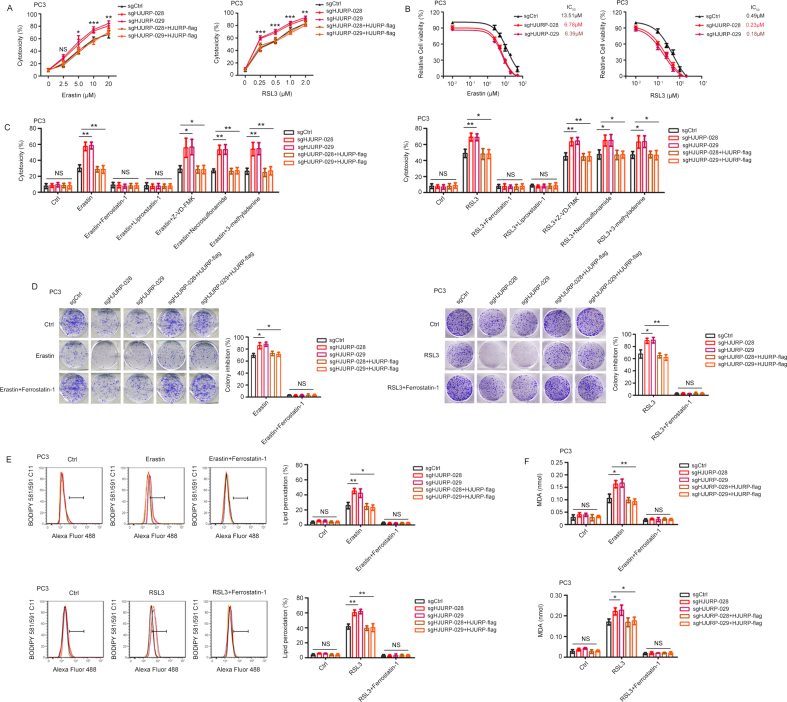


**figs3:**
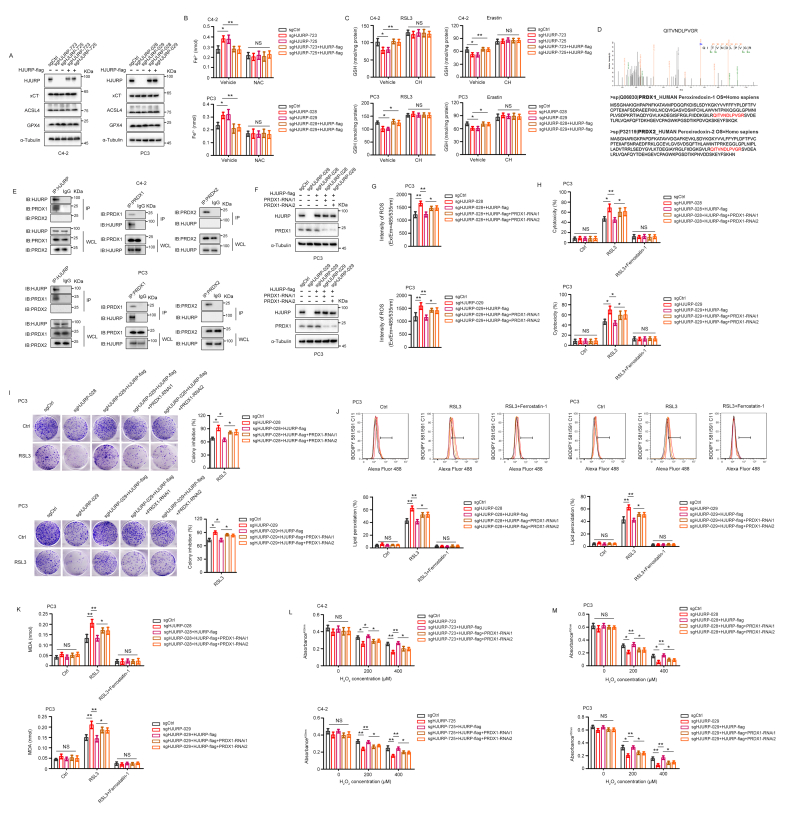


**figs4:**
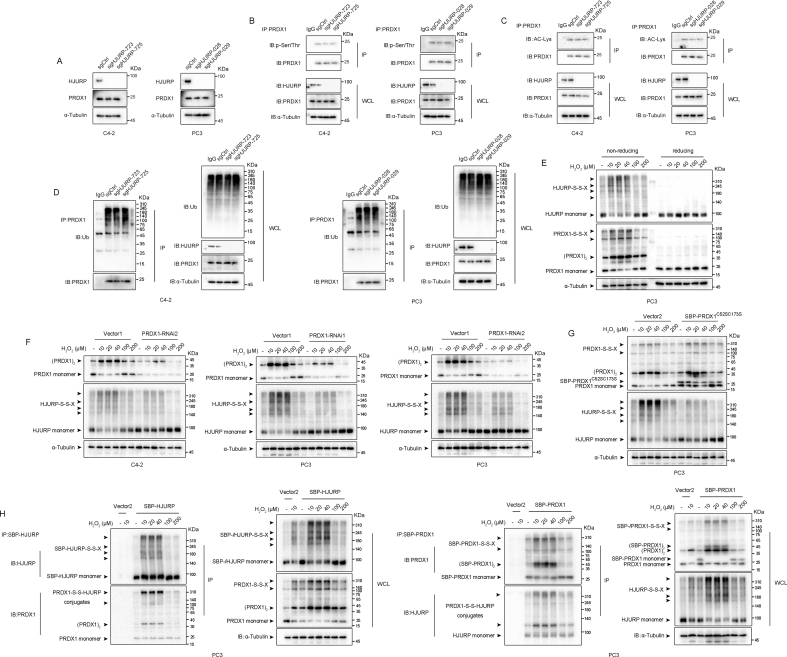


**figs5:**
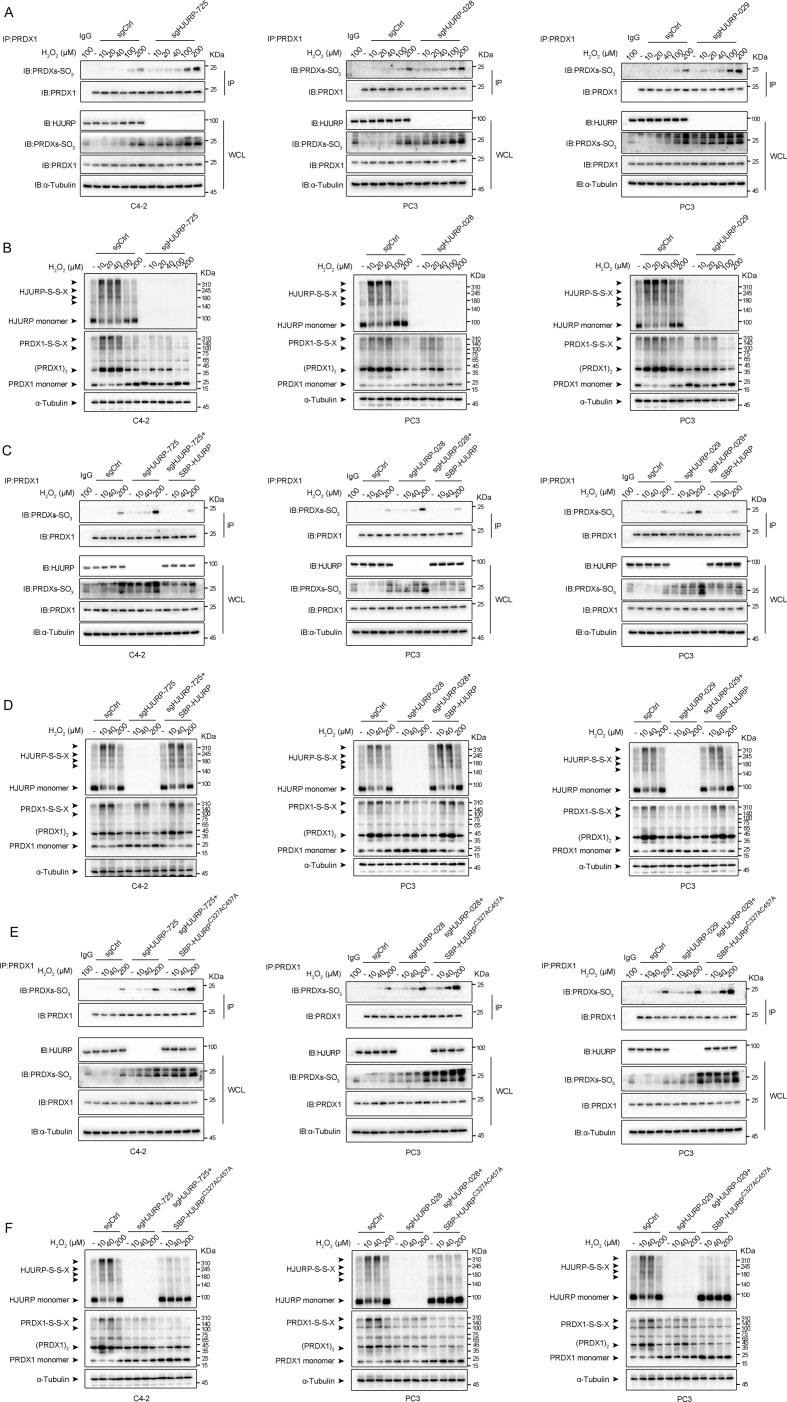


**figs6:**
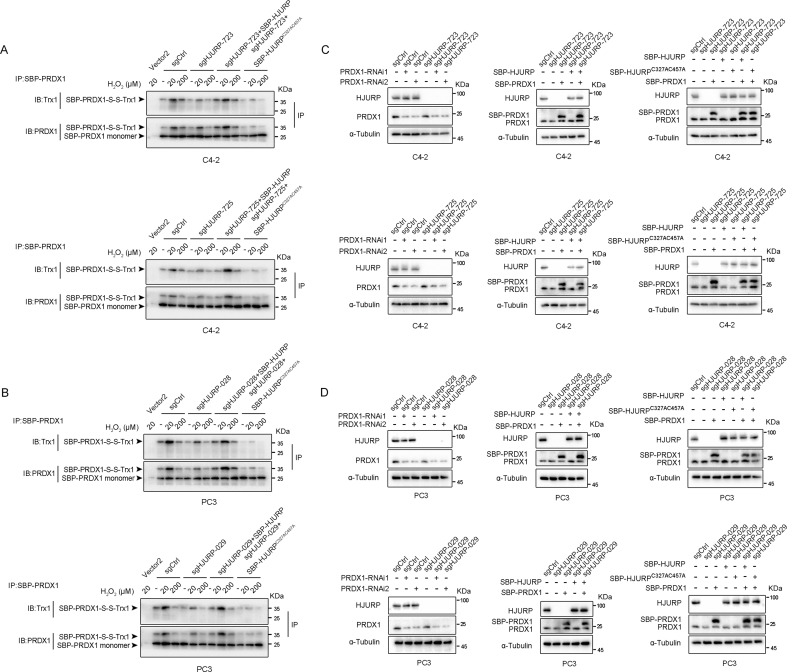


**figs7:**
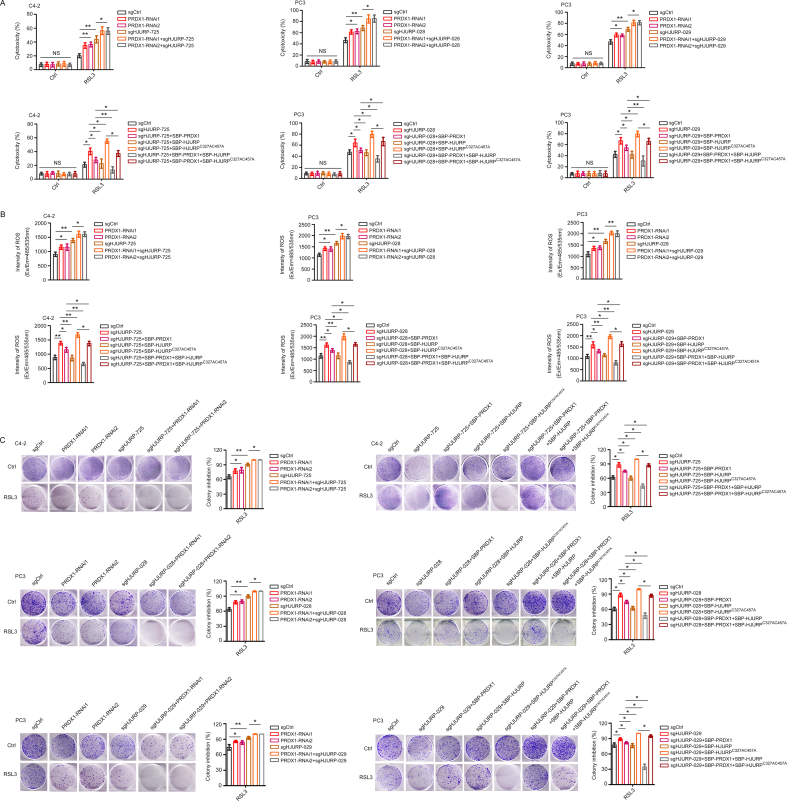


**figs8:**
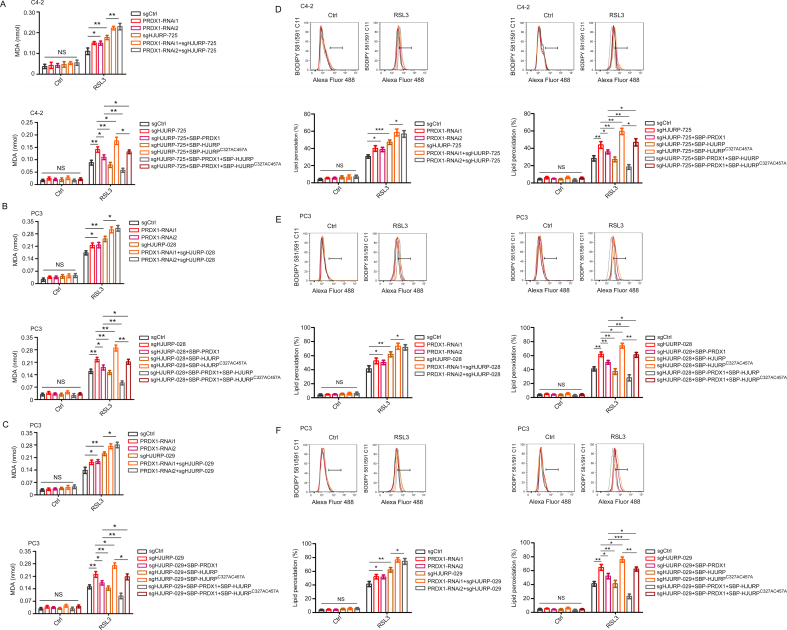


## Data Availability

Data will be made available on request.

## References

[1] Sung H., Ferlay J., Siegel R.L., Laversanne M., Soerjomataram I., Jemal A. (2021). Global cancer statistics 2020: GLOBOCAN estimates of incidence and mortality worldwide for 36 cancers in 185 countries. CA A Cancer J. Clin..

[2] Siegel R.L., Miller K.D., Fuchs H.E., Jemal A. (2022). Cancer statistics, 2022. CA A Cancer J. Clin..

[3] Takahashi T. (2023). Prostate cancer screening and the golden rule of humanity. BMJ.

[4] Preisser F., Abrams-Pompe R.S., Stelwagen P.J., Böhmer D., Zattoni F., Magli A. (2023). European association of urology biochemical recurrence risk classification as a decision tool for salvage radiotherapy-a multicenter study. Eur. Urol..

[5] Sandhu S., Moore C.M., Chiong E., Beltran H., Bristow R.G., Williams S.G. (2021). Prostate cancer. Lancet..

[6] Gravis G. (2023). Metastatic prostate cancer management: 20 years of progress. Lancet Oncol..

[7] Teo M.Y., Rathkopf D.E., Kantoff P. (2019). Treatment of advanced prostate cancer. Annu. Rev. Med..

[8] Liang D., Feng Y., Zandkarimi F., Wang H., Zhang Z., Kim J. (2023). Ferroptosis surveillance independent of GPX4 and differentially regulated by sex hormones. Cell.

[9] Wang M.E., Chen J., Lu Y., Bawcom A.R., Wu J., Ou J. (2023). RB1-deficient prostate tumor growth and metastasis are vulnerable to ferroptosis induction via the E2F/ACSL4 axis. J. Clin. Invest..

[10] Wang J., Zeng L., Wu N., Liang Y., Jin J., Fan M. (2023). Inhibition of phosphoglycerate dehydrogenase induces ferroptosis and overcomes enzalutamide resistance in castration-resistant prostate cancer cells. Drug Resist. Updates.

[11] Viswanathan V.S., Ryan M.J., Dhruv H.D., Gill S., Eichhoff O.M., Seashore-Ludlow B. (2017). Dependency of a therapy-resistant state of cancer cells on a lipid peroxidase pathway. Nature.

[12] Ghoochani A., Hsu E.C., Aslan M., Rice M.A., Nguyen H.M., Brooks J.D. (2021). Ferroptosis inducers are a novel therapeutic approach for advanced prostate cancer. Cancer Res..

[13] Mao M., Jia Y., Chen Y., Yang J., Xu L., Zhang X. (2022). HJURP regulates cell proliferation and chemo-resistance via YAP1/NDRG1 transcriptional axis in triple-negative breast cancer. Cell Death Dis..

[14] Jia Y., Zhou J., Tan T.K., Chung T.H., Chen Y., Chooi J.Y. (2022). Super enhancer-mediated upregulation of HJURP promotes growth and survival of t(4;14)-positive multiple myeloma. Cancer Res..

[15] Wang C.J., Li X., Shi P., Ding H.Y., Liu Y.P., Li T. (2020). Holliday junction recognition protein promotes pancreatic cancer growth and metastasis via modulation of the MDM2/p53 signaling. Cell Death Dis..

[16] Lai W., Zhu W., Xiao C., Li X., Wang Y., Han Y. (2021). HJURP promotes proliferation in prostate cancer cells through increasing CDKN1A degradation via the GSK3β/JNK signaling pathway. Cell Death Dis..

[17] Li L., Yuan Q., Chu Y.M., Jiang H.Y., Zhao J.H., Su Q., Huo D.Q., Zhang X.F. (2023 Mar 21). Advances in holliday junction recognition protein (HJURP): structure, molecular functions, and roles in cancer. Front. Cell Dev. Biol..

[18] Stockwell B.R. (2022). Ferroptosis turns 10: emerging mechanisms, physiological functions, and therapeutic applications. Cell.

[19] Liu T., Sun L., Zhang Y., Wang Y., Zheng J. (2022). Imbalanced GSH/ROS and sequential cell death. J. Biochem. Mol. Toxicol..

[20] Kakhlon O., Cabantchik Z.I. (2002). The labile iron pool: characterization, measurement, and participation in cellular processes(1). Free Radic. Biol. Med..

[21] Liu Y., Wang P., Hu W., Chen D. (2023). New insights into the roles of peroxiredoxins in cancer. Biomed. Pharmacother..

[22] Woo H.A., Yim S.H., Shin D.H., Kang D., Yu D.Y., Rhee S.G. (2010). Inactivation of peroxiredoxin I by phosphorylation allows localized H_2_O_2_ accumulation for cell signaling. Cell.

[23] Choi H., Kim H.J., Kim J., Kim S., Yang J., Lee W. (2017). Increased acetylation of Peroxiredoxin1 by HDAC6 inhibition leads to recovery of Aβ-induced impaired axonal transport. Mol. Neurodegener..

[24] Min Y., Kim M.J., Lee S., Chun E., Lee K.Y. (2018). Inhibition of TRAF6 ubiquitin-ligase activity by PRDX1 leads to inhibition of NFKB activation and autophagy activation. Autophagy.

[25] Ding C., Fan X., Wu G. (2017). Peroxiredoxin 1 - an antioxidant enzyme in cancer. J. Cell Mol. Med..

[26] Kim Y., Jang H.H. (2019). Role of cytosolic 2-Cys prx1 and prx2 in redox signaling. Antioxidants.

[27] Dasari C., Reddy K., Natani S., Murthy T., Bhukya S., Ummanni R. (2019). Tumor protein D52 (isoform 3) interacts with and promotes peroxidase activity of Peroxiredoxin 1 in prostate cancer cells implicated in cell growth and migration. Biochim. Biophys. Acta Mol. Cell Res..

[28] Jarvis R.M., Hughes S.M., Ledgerwood E.C. (2012). Peroxiredoxin 1 functions as a signal peroxidase to receive, transduce, and transmit peroxide signals in mammalian cells. Free Radic. Biol. Med..

[29] Neumann C.A., Cao J., Manevich Y. (2009). Peroxiredoxin 1 and its role in cell signaling. Cell Cycle.

[30] Oh M., Jang S.Y., Lee J.Y., Kim J.W., Jung Y., Kim J. (2023). The lipoprotein-associated phospholipase A2 inhibitor Darapladib sensitises cancer cells to ferroptosis by remodelling lipid metabolism. Nat. Commun..

[31] Lei G., Zhuang L., Gan B. (2022). Targeting ferroptosis as a vulnerability in cancer. Nat. Rev. Cancer.

[32] Jiang X., Stockwell B.R., Conrad M. (2021). Ferroptosis: mechanisms, biology and role in disease. Nat. Rev. Mol. Cell Biol..

[33] Dixon S.J., Lemberg K.M., Lamprecht M.R., Skouta R., Zaitsev E.M., Gleason C.E. (2012). Ferroptosis: an iron-dependent form of nonapoptotic cell death. Cell.

[34] Papavasileiou G., Tsilingiris D., Spyrou N., Vallianou N.G., Karampela I., Magkos F. (2023). Obesity and main urologic cancers: current systematic evidence, novel biological mechanisms, perspectives and challenges. Semin. Cancer Biol..

[35] Nassar Z.D., Mah C.Y., Dehairs J., Burvenich I.J., Irani S., Centenera M.M. (2020). Human DECR1 is an androgen-repressed survival factor that regulates PUFA oxidation to protect prostate tumor cells from ferroptosis. Elife.

[36] Blomme A., Peter C., Mui E., Rodriguez Blanco G., An N., Mason L.M. (2022). THEM6-mediated reprogramming of lipid metabolism supports treatment resistance in prostate cancer. EMBO Mol. Med..

[37] Chen X., Kang R., Kroemer G., Tang D. (2021). Broadening horizons: the role of ferroptosis in cancer. Nat. Rev. Clin. Oncol..

[38] Biteau B., Labarre J., Toledano M.B. (2003). ATP-dependent reduction of cysteine-sulphinic acid by S. cerevisiae sulphiredoxin. Nature.

[39] Yang K.S., Kang S.W., Woo H.A., Hwang S.C., Chae H.Z., Kim K. (2002). Inactivation of human peroxiredoxin I during catalysis as the result of the oxidation of the catalytic site cysteine to cysteine-sulfinic acid. J. Biol. Chem..

[40] Yilmaz D., Furst A., Meaburn K., Lezaja A., Wen Y., Altmeyer M. (2021). Activation of homologous recombination in G1 preserves centromeric integrity. Nature.

[41] Cao R., Wang G., Qian K., Chen L., Qian G., Xie C. (2017). Silencing of HJURP induces dysregulation of cell cycle and ROS metabolism in bladder cancer cells via PPARγ-SIRT1 feedback loop. J. Cancer.

[42] Yuan J.S., Chen Z.S., Wang K., Zhang Z.L. (2020). Holliday junction-recognition protein modulates apoptosis, cell cycle arrest and reactive oxygen species stress in human renal cell carcinoma. Oncol. Rep..

[43] de Tayrac M., Saikali S., Aubry M., Bellaud P., Boniface R., Quillien V. (2013). Prognostic significance of EDN/RB, HJURP, p60/CAF-1 and PDLI4, four new markers in high-grade gliomas. PLoS One.

[44] Kang D.H., Woo J., Kim H., Kim S.Y., Ji S., Jaygal G. (2020). Prognostic relevance of HJURP expression in patients with surgically resected colorectal cancer. Int. J. Mol. Sci..

[45] Murphy M.P., Bayir H., Belousov V., Chang C.J., Davies K.J.A., Davies M.J. (2022). Guidelines for measuring reactive oxygen species and oxidative damage in cells and in vivo. Nat. Metab..

