# Corrigendum to “HJURP inhibits sensitivity to ferroptosis inducers in prostate cancer cells by enhancing the peroxidase activity of PRDX1” [Redox Biology 77 (2024) 103392]

**DOI:** 10.1016/j.redox.2026.104159

**Published:** 2026-04-08

**Authors:** Wenjie Lai, Weian Zhu, Jianjie Wu, Jiongduan Huang, Xiaojuan Li, Yun Luo, Yu Wang, Hengda Zeng, Mingqiang Li, Xiaofu Qiu, Xingqiao Wen

**Affiliations:** a Department of Urology, The Affiliated Guangdong Second Provincial General Hospital of Jinan University, Guangzhou, Guangdong, 510317, PR China; b Department of Urology, The Third Affiliated Hospital, Sun Yat-sen University, Guangzhou, Guangdong, 510630, PR China; c Department of Urology, Shenshan Medical Center, Memorial Hospital of Sun Yat-sen University, Shanwei, Guangdong, 516600, PR China; d Department of Health Care, Shenzhen Hospital, Southern Medical University, Shenzhen, Guangdong, 518101, PR China; e Laboratory of Biomaterials and Translational Medicine, The Third Affiliated Hospital, Sun Yat-sen University, Guangzhou, Guangdong, 510630, PR China; f Department of Urology, Guangdong Provincial People's Hospital（Guangdong Academy of Medical Sciences）, Southern Medical University, Guangzhou, 510080, PR China

The authors request an addition of “Guangdong Academy of Medical Sciences” to the existing affiliation of the corresponding author Xingqiao Wen.

The updated affiliation of Xingqiao Wen is: Department of Urology, Guangdong Provincial People's Hospital (Guangdong Academy of Medical Sciences), Southern Medical University, Guangzhou, 510080, PR China.

The authors would like to apologise for any inconvenience caused.

