# Supplementary material for: HJURP inhibits sensitivity to ferroptosis inducers in prostate cancer cells by enhancing the peroxidase activity of PRDX1
Source: Redox Biol. 2024 Oct 10;77:103392. doi: 10.1016/j.redox.2024.103392 (PMC11525750; doi:10.1016/j.redox.2024.103392)
Supplement: Multimedia component 9 [file mmc9.pdf]

Fig.2C & Fig.S3E

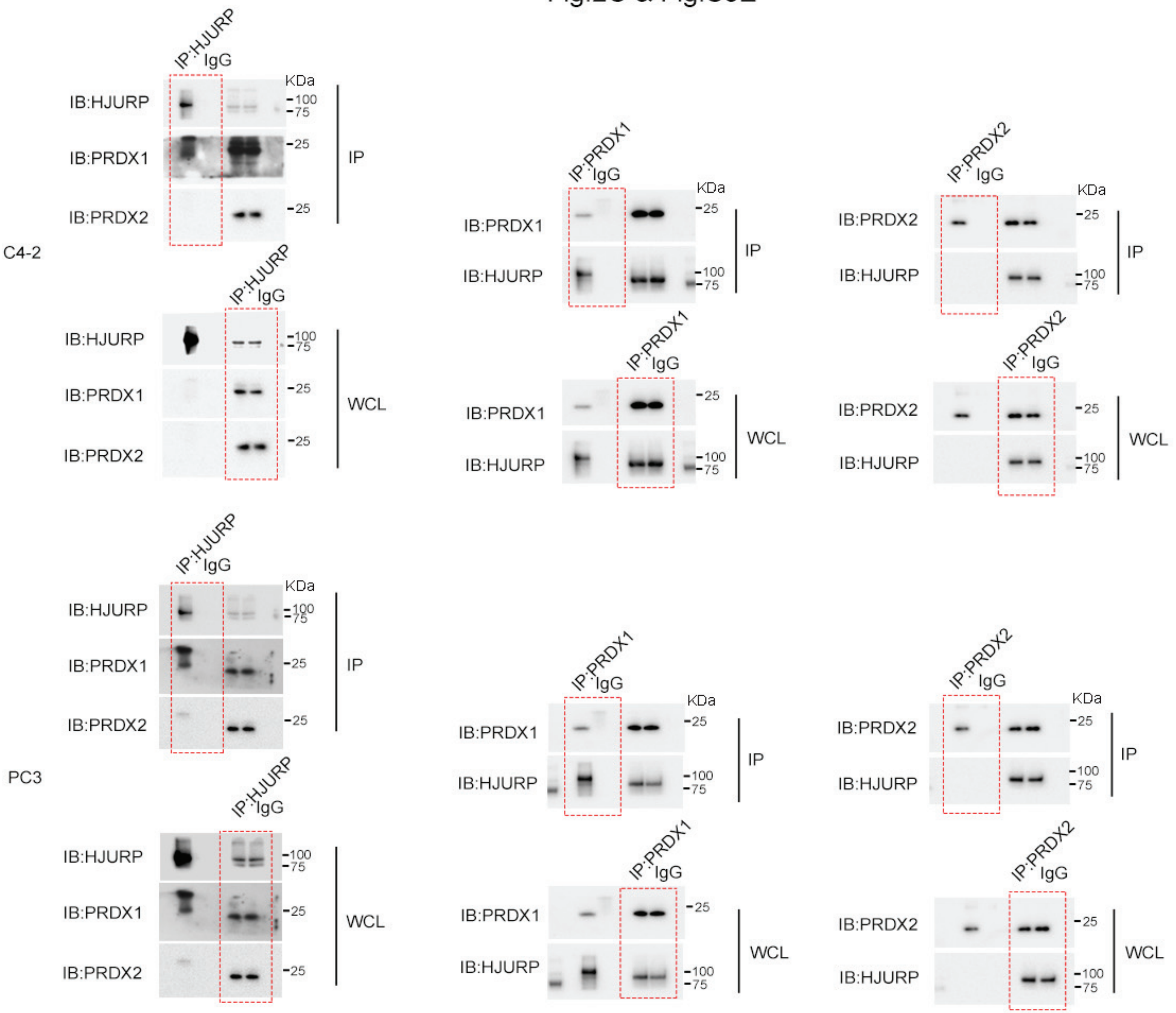

Fig.3A

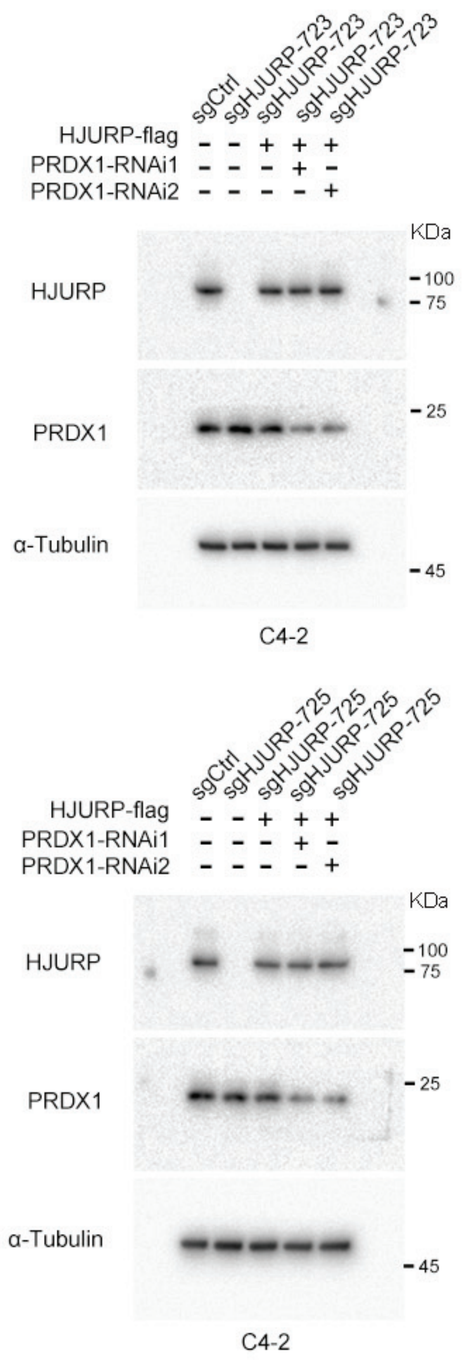

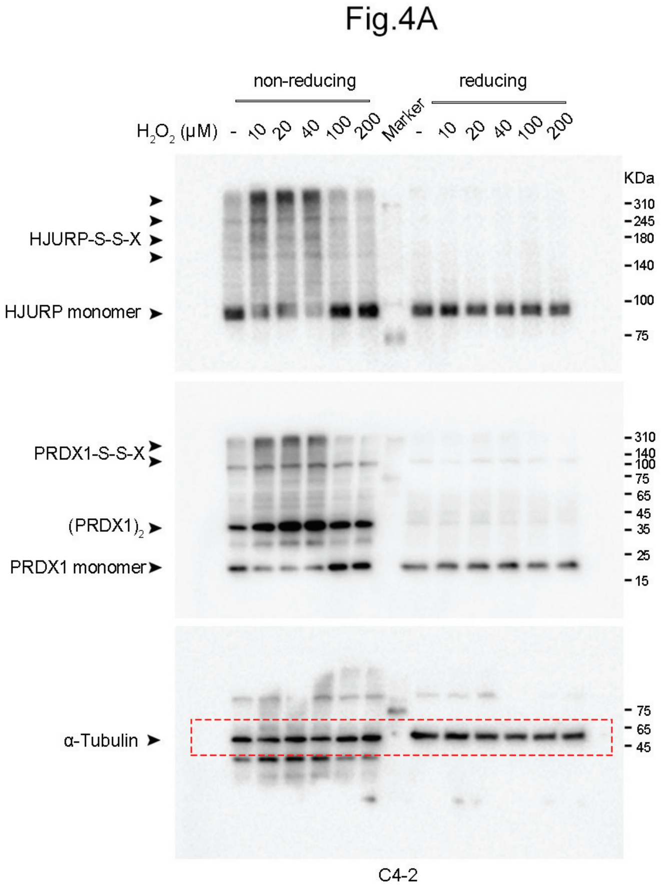

Fig.4A
non-reducing
reducing
H2O2 (μM)
- 10 20 40 100 200 Marker - 10 20 40 100 200
KDa
HJURP-S-S-X
HJURP monomer
PRDX1-S-S-X
(PRDX1)2
PRDX1 monomer
α-Tubulin
310
245
180
140
100
75
65
45
35
25
15
C4-2

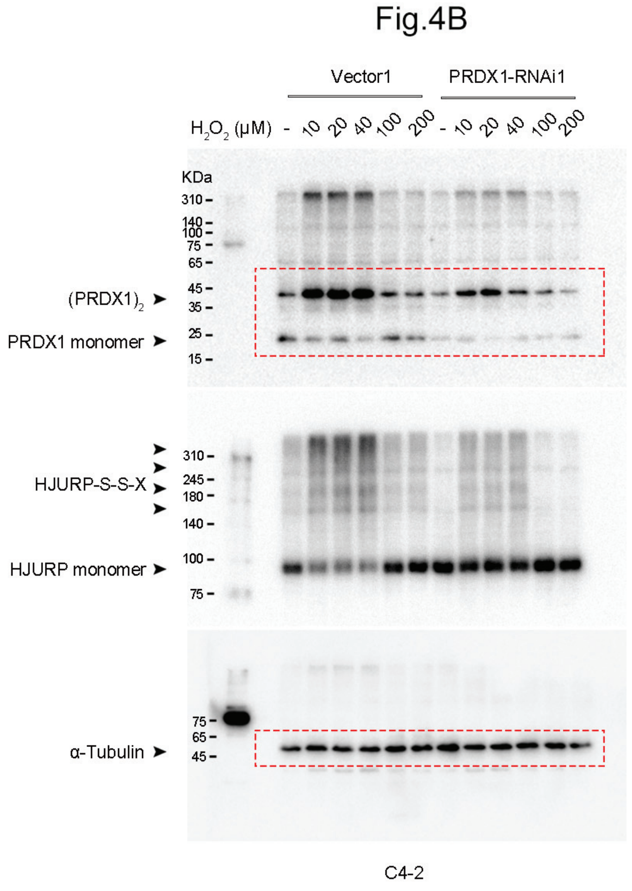

Fig.4B
Vector1
PRDX1-RNAi1
H2O2 (μM)
- 10 20 40 100 200 - 10 20 40 100 200
KDa
(PRDX1)2
PRDX1 monomer
HJURP-S-S-X
HJURP monomer
α-Tubulin
310
245
180
140
100
75
65
45
35
25
15
C4-2

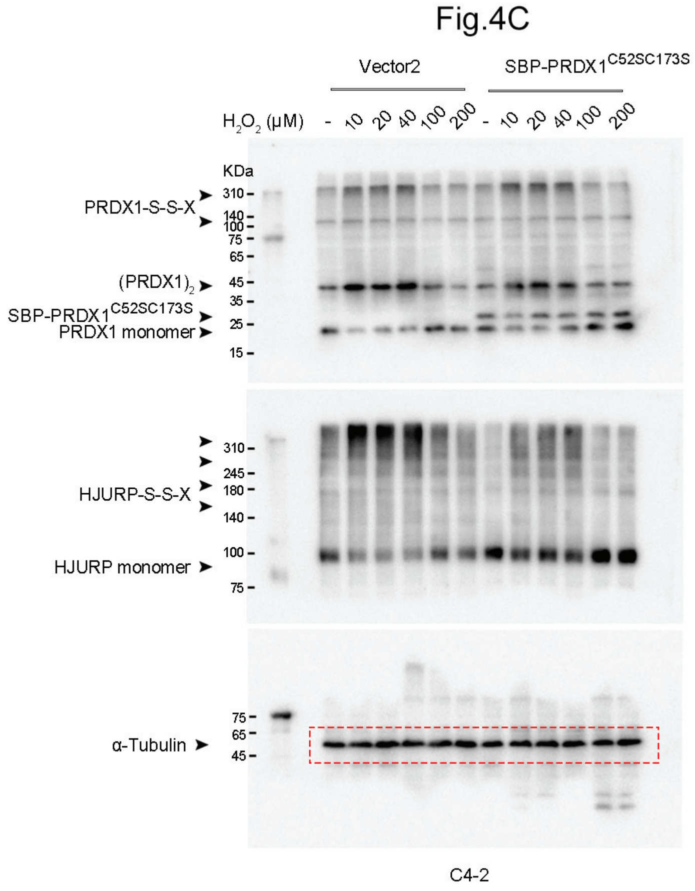

Fig.4C
Vector2
SBP-PRDX1C52SC173S
H2O2 (μM)
- 10 20 40 100 200 - 10 20 40 100 200
KDa
PRDX1-S-S-X
(PRDX1)2
SBP-PRDX1C52SC173S
PRDX1 monomer
HJURP-S-S-X
HJURP monomer
α-Tubulin
310
245
180
140
100
75
65
45
35
25
15
C4-2

Fig.4D

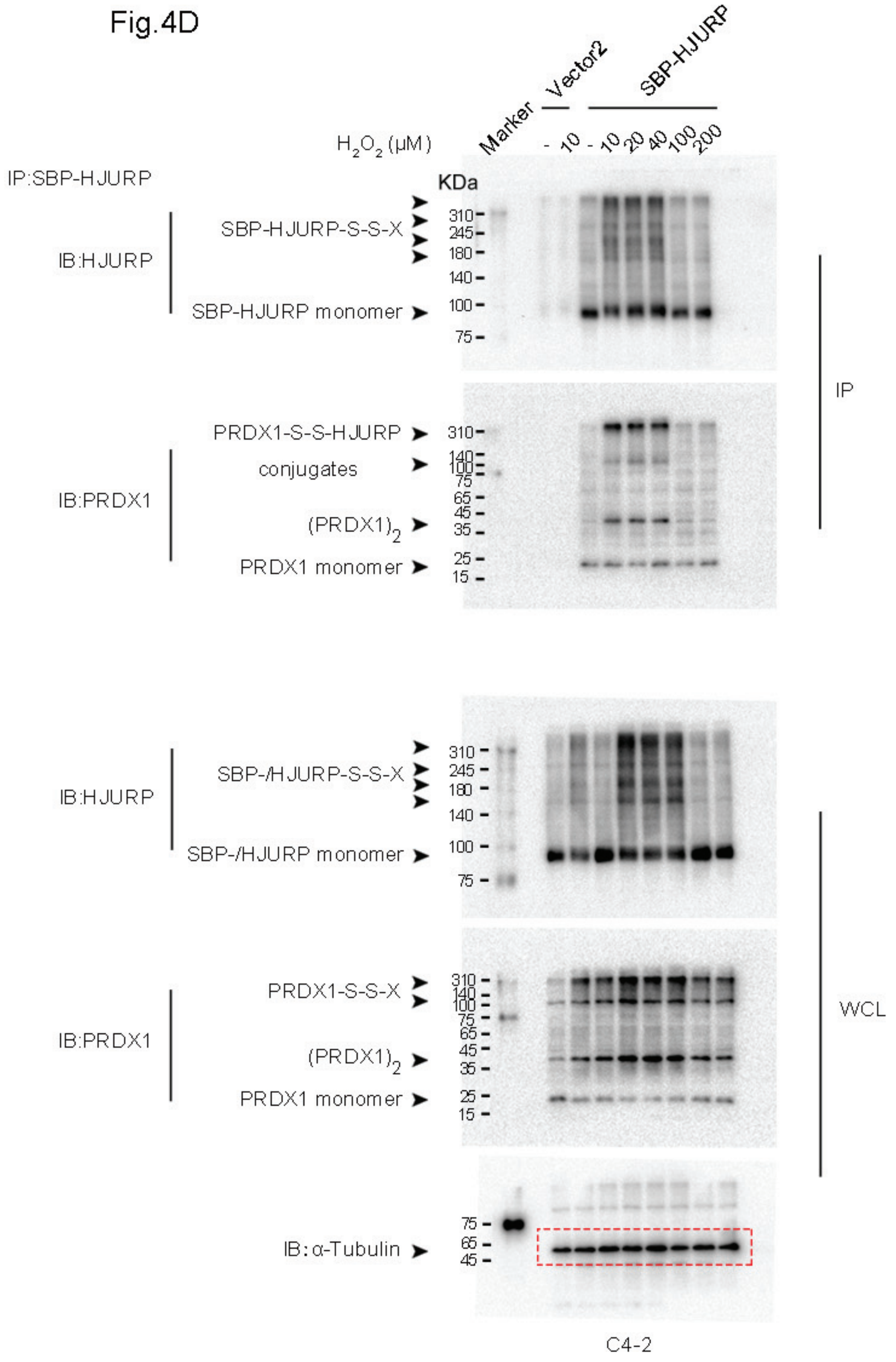

Marker
Vector2
SBP-HJURP
H2O2 (μM)
- 10 - 10 20 40 100 200
IP:SBP-HJURP
KDa
IB:HJURP
SBP-HJURP-S-S-X
SBP-HJURP monomer
IB:PRDX1
PRDX1-S-S-HJURP
conjugates
(PRDX1)2
PRDX1 monomer
IP
IB:HJURP
SBP-/HJURP-S-S-X
SBP-/HJURP monomer
IB:PRDX1
PRDX1-S-S-X
(PRDX1)2
PRDX1 monomer
WCL
IB: α-Tubulin
C4-2

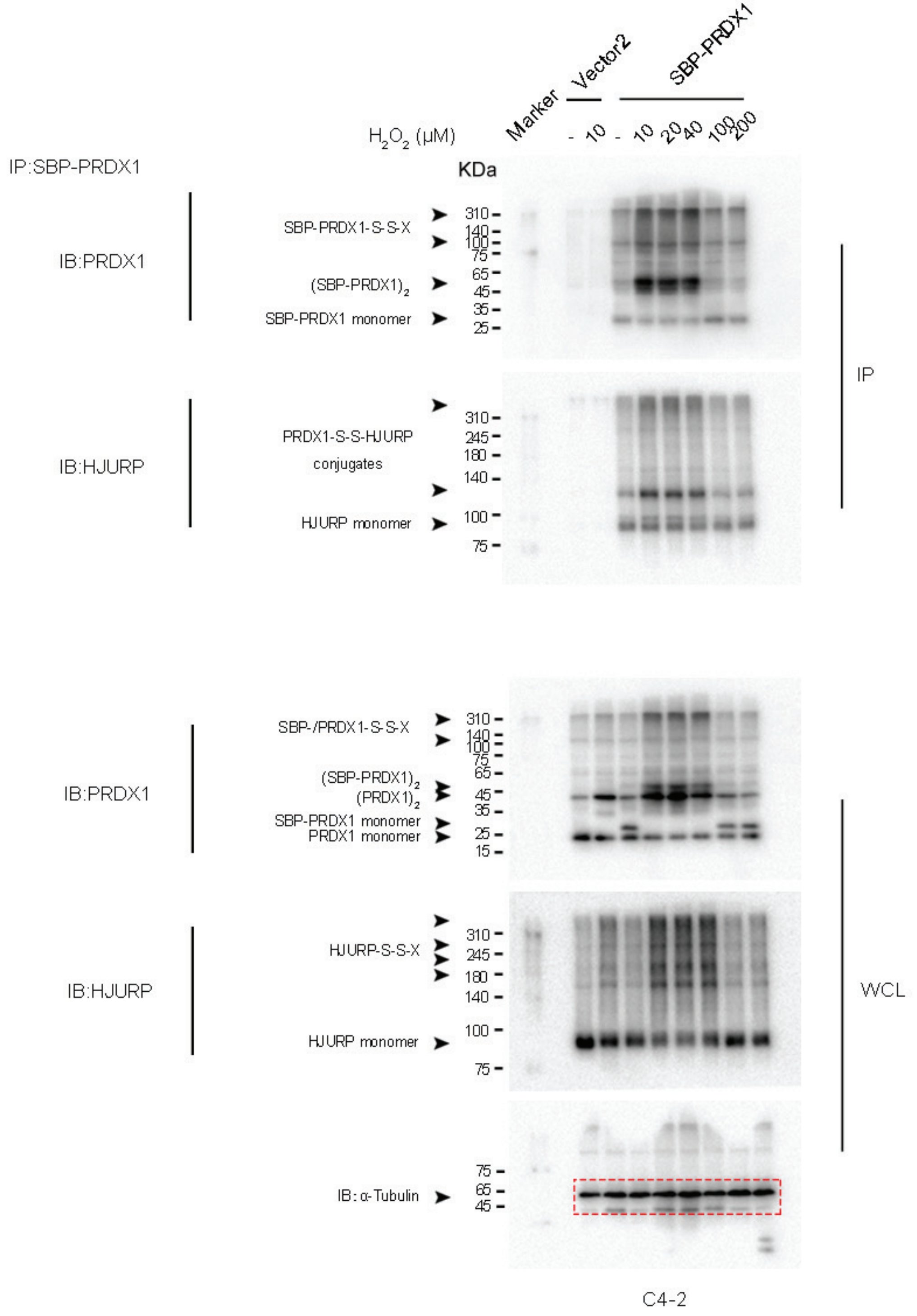

Marker
Vector2
SBP-PRDX1
H2O2 (μM)
- 10 - 10 20 40 100 200
IP:SBP-PRDX1
KDa
IB:PRDX1
SBP-PRDX1-S-S-X
(SBP-PRDX1)2
SBP-PRDX1 monomer
IB:HJURP
PRDX1-S-S-HJURP
conjugates
HJURP monomer
IP
IB:PRDX1
SBP-/PRDX1-S-S-X
(SBP-PRDX1)2
(PRDX1)2
SBP-PRDX1 monomer
PRDX1 monomer
IB:HJURP
HJURP-S-S-X
HJURP monomer
WCL
IB: α-Tubulin
C4-2

Fig.4F

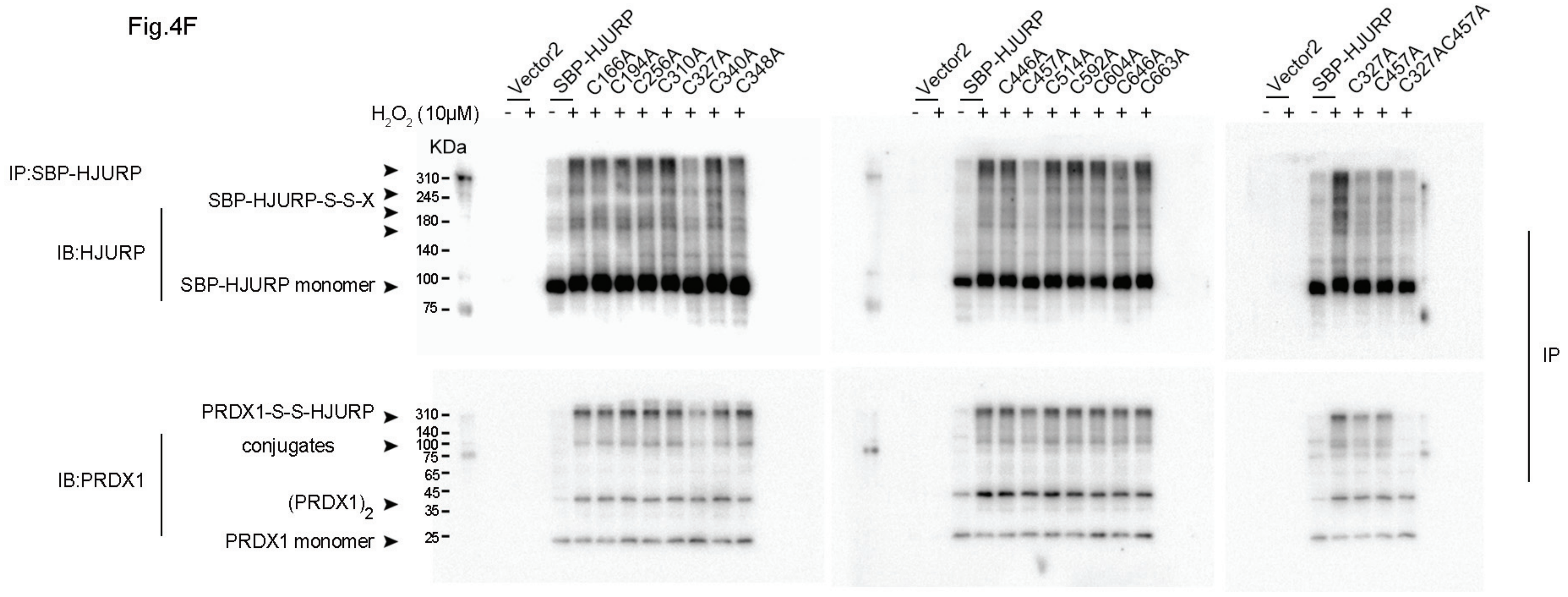
Vector2
SBP-HJURP
C166A
C194A
C256A
C310A
C327A
C340A
C348A
C446A
C457A
C514A
C592A
C604A
C646A
C663A
C327AC457A
H_2O_2 (10μM)
KDa
IP:SBP-HJURP
SBP-HJURP-S-S-X
IB:HJURP
SBP-HJURP monomer
310
245
180
140
100
75
PRDX1-S-S-HJURP
conjugates
IB:PRDX1
65
45
35
25
$(PRDX1)_2$
PRDX1 monomer
IP

C4-2

Fig.5A

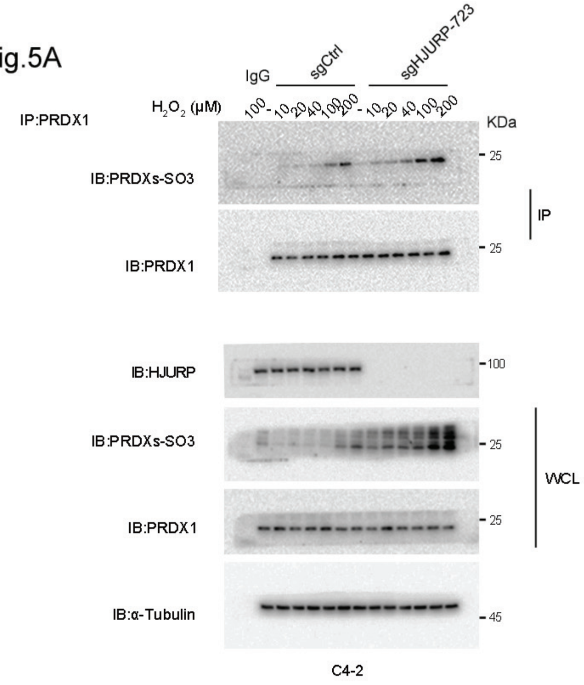

Fig.5C

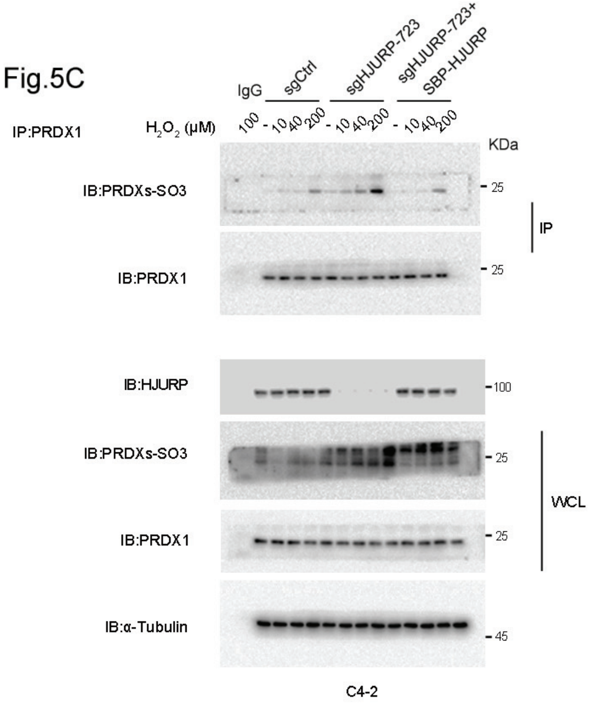

Fig.5E

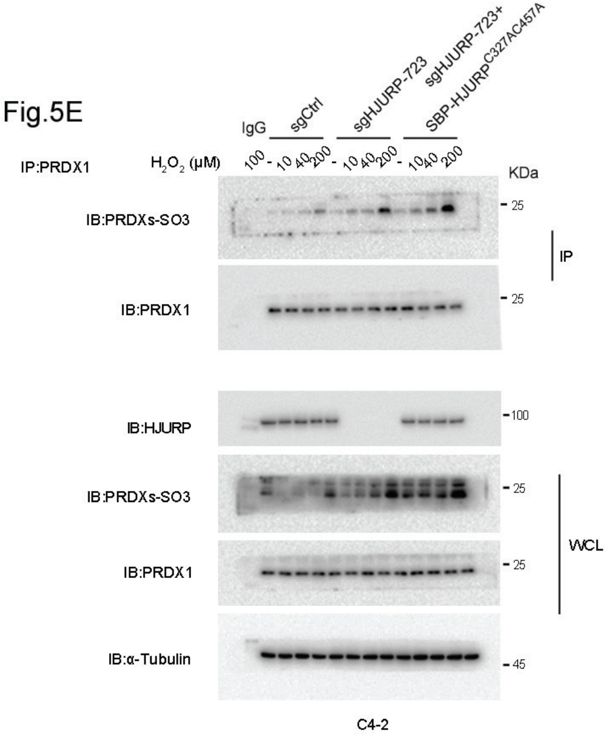

Fig.5B

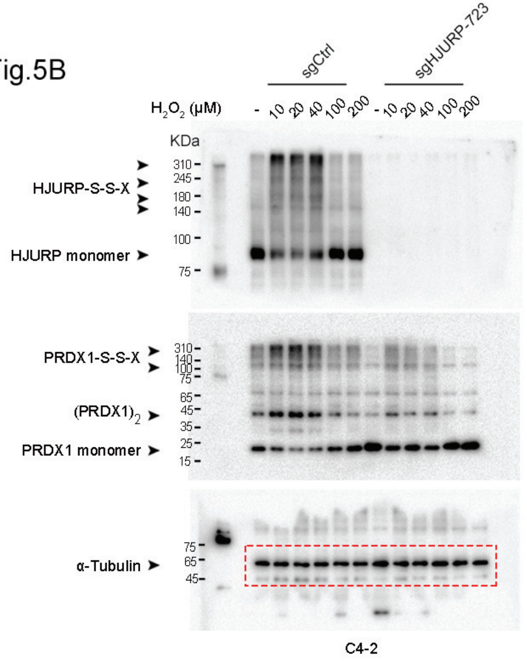

Fig.5D

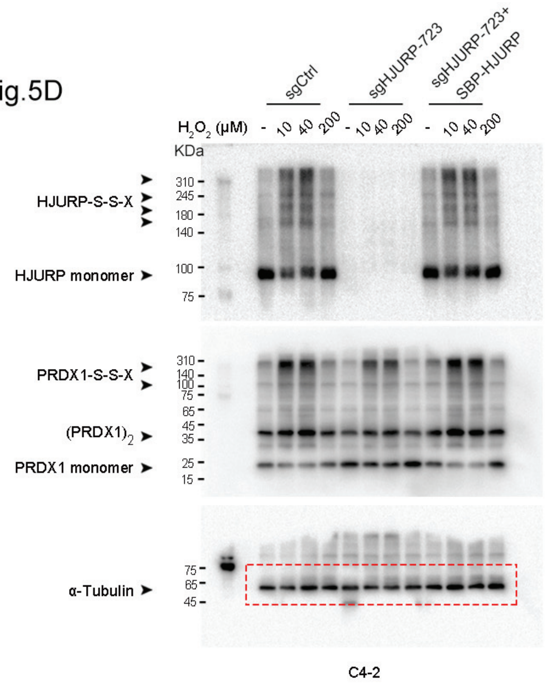

Fig.5F

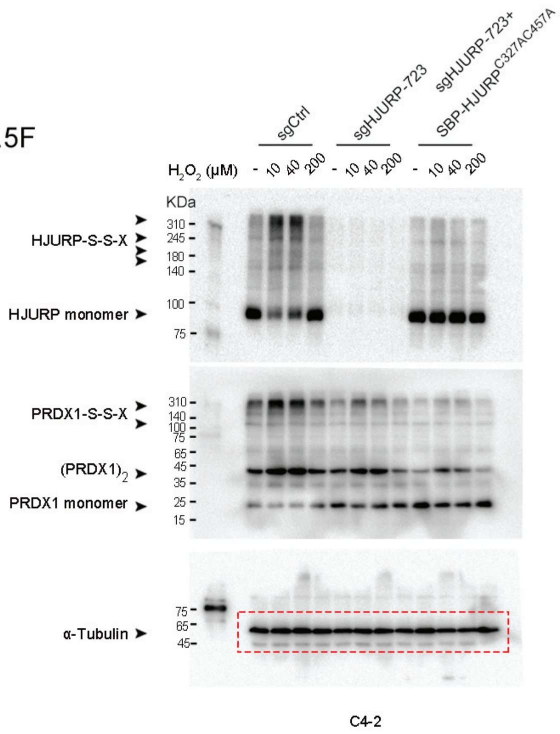

Fig.S1A

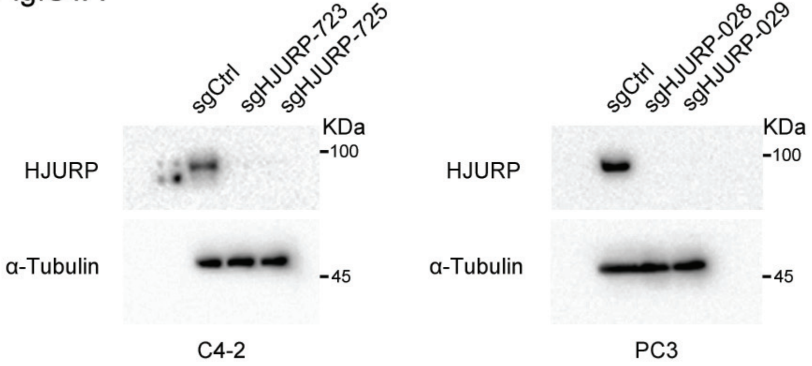

Fig.S1H

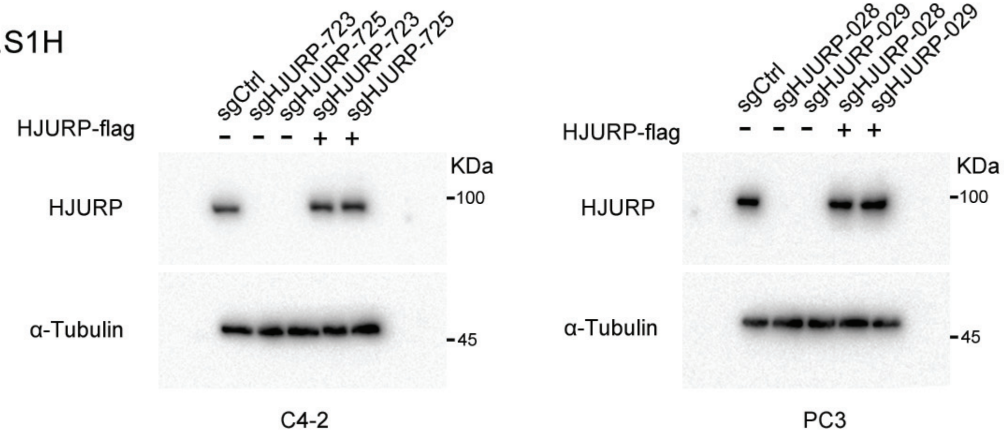

Fig.S3A

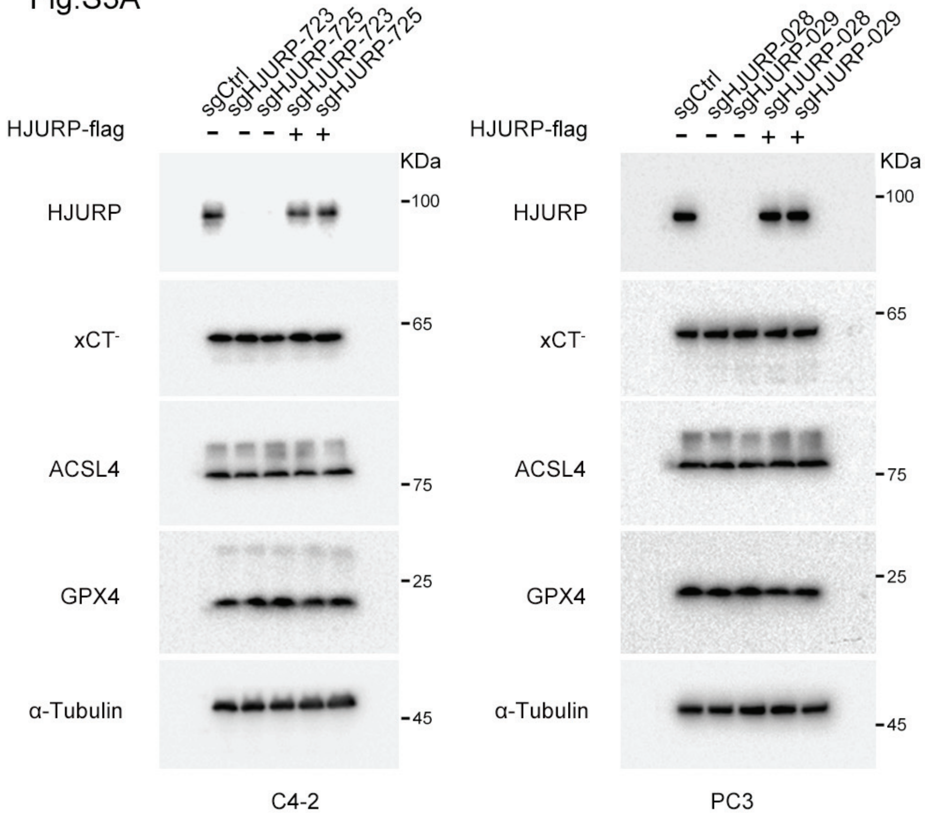

Fig.S3F

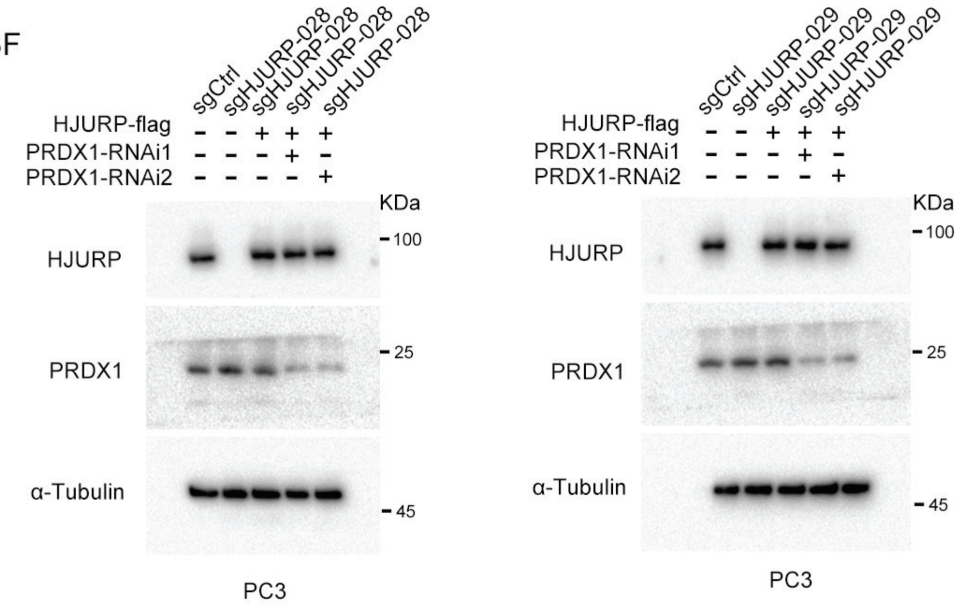

Fig.S4A

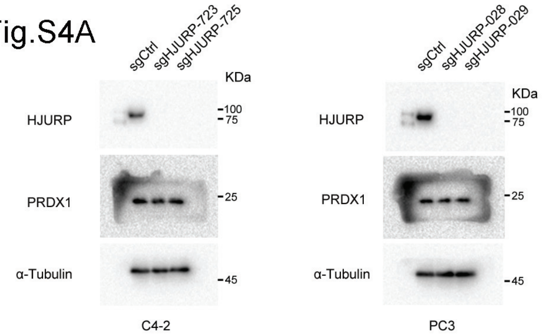

Fig.S4B

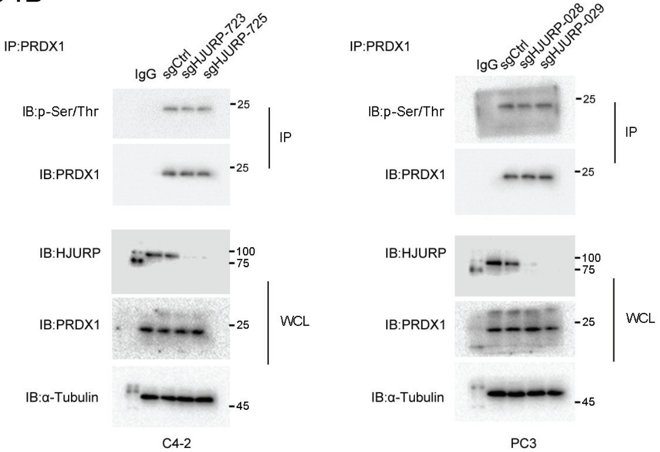

Fig.S4C

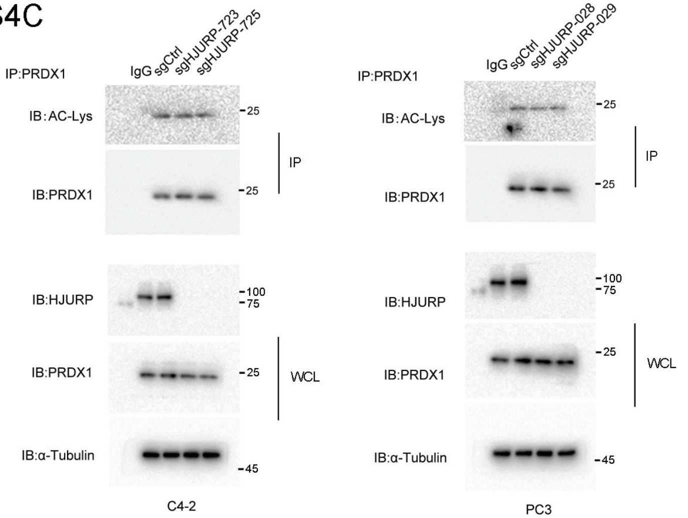

Fig.S4D

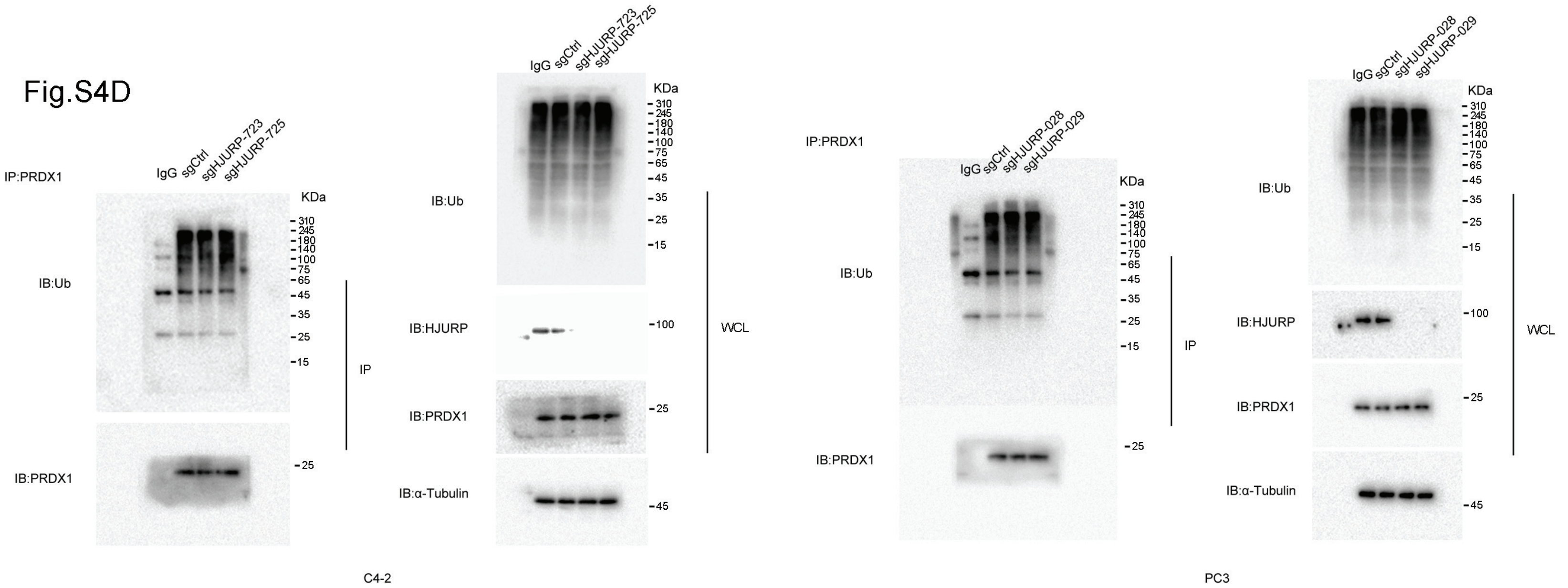

Fig.S4E

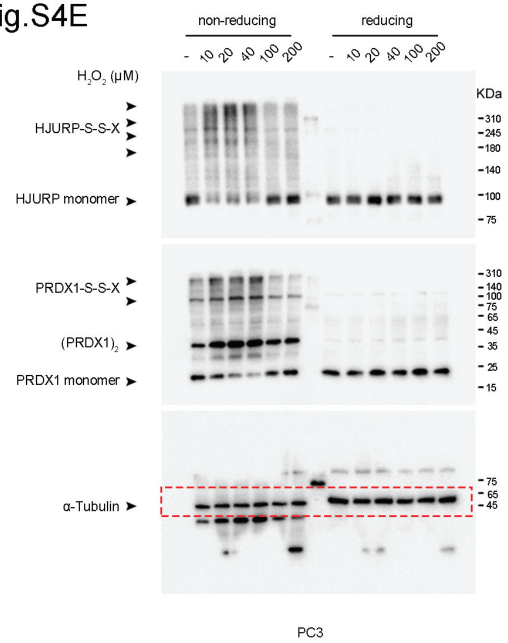

Fig.S4F

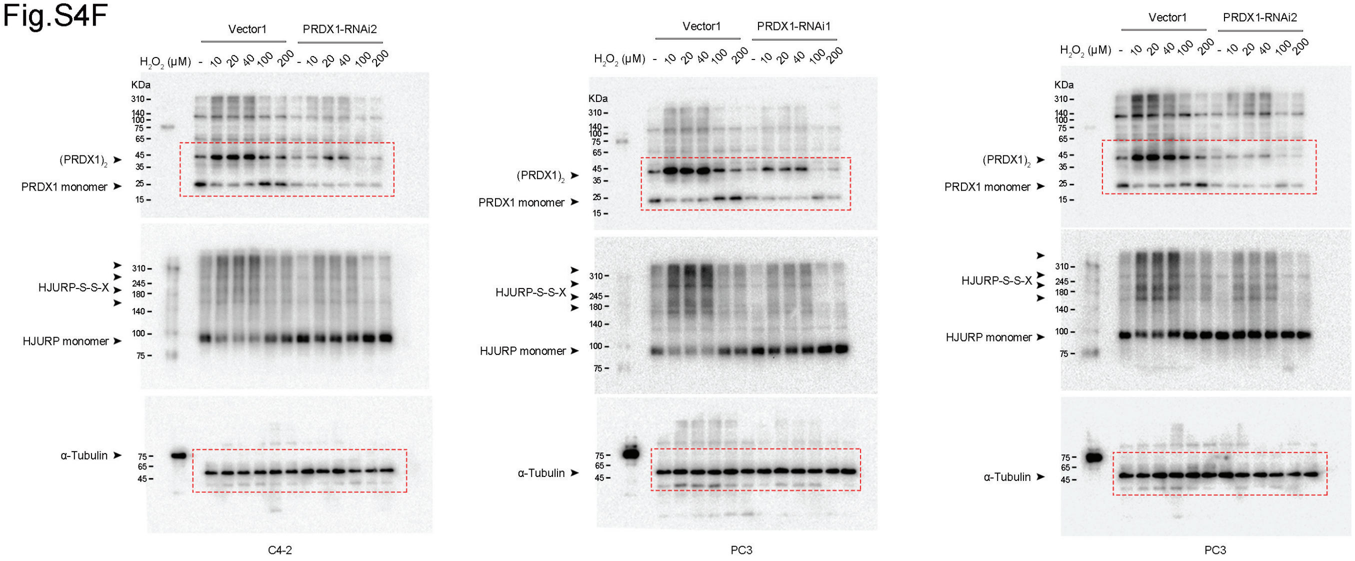

Fig.S4G

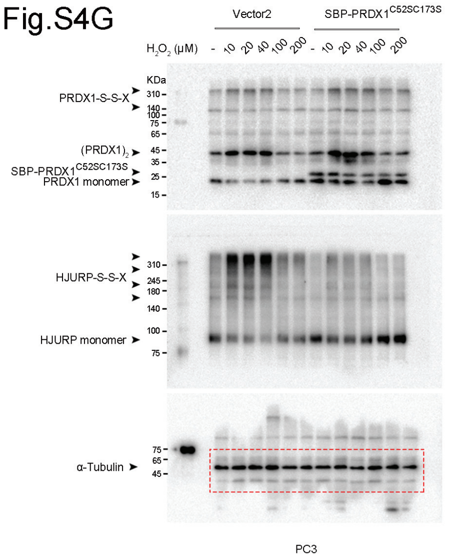

Fig.S4H

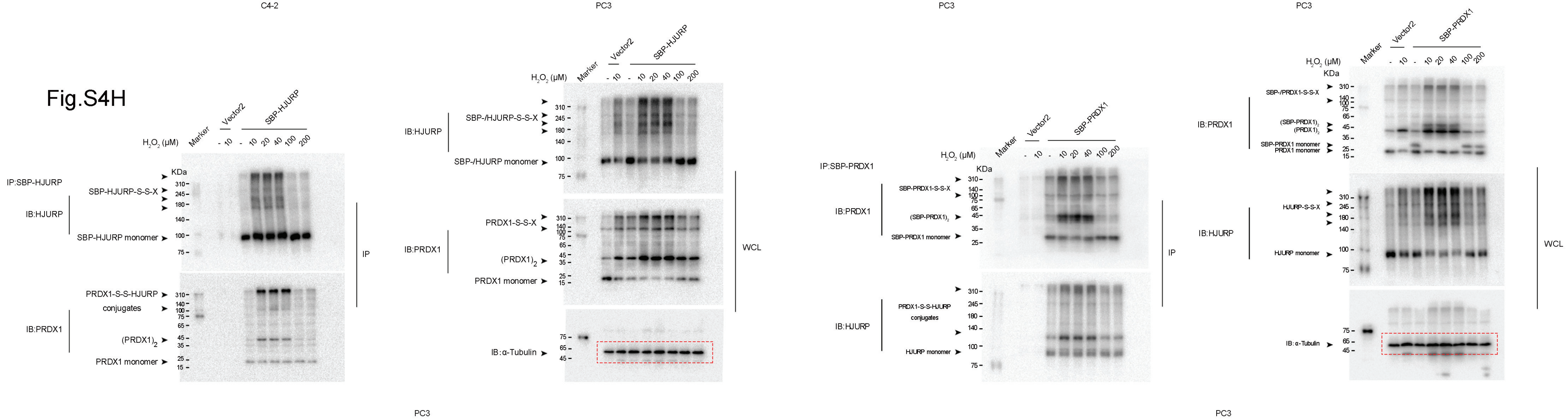

Fig.S5A

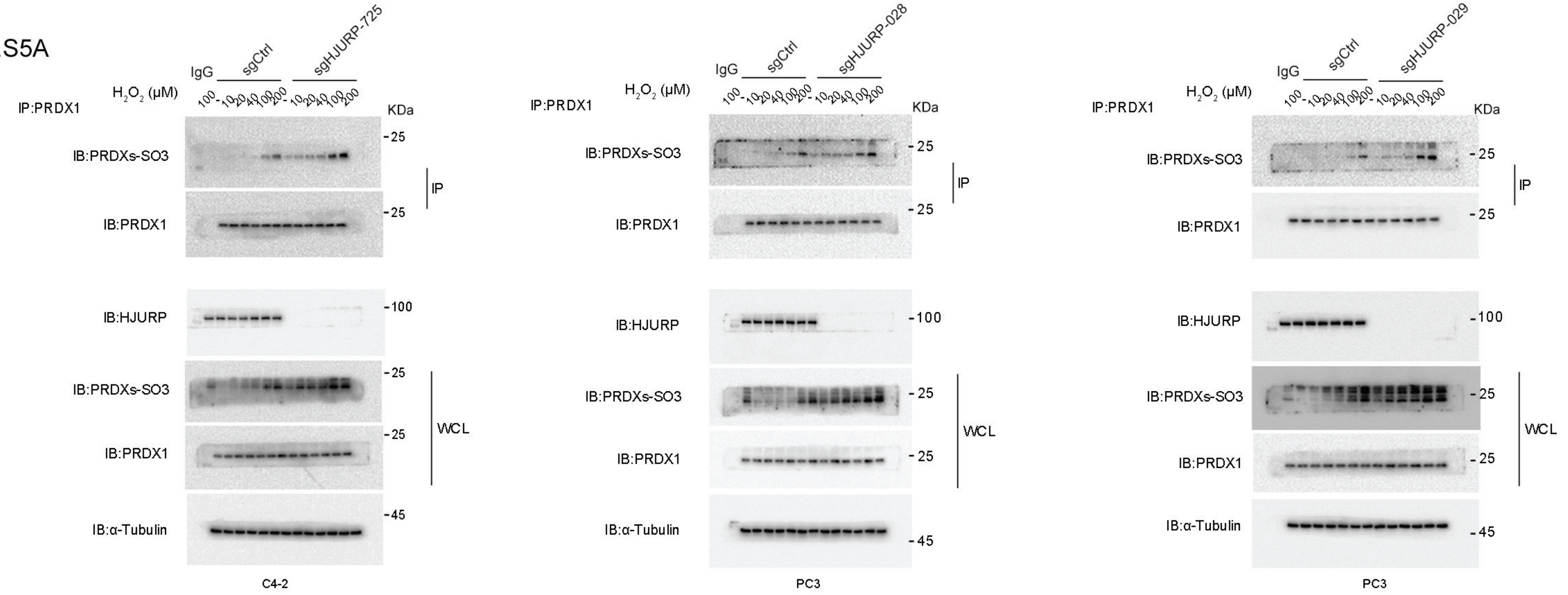

Fig.S5B

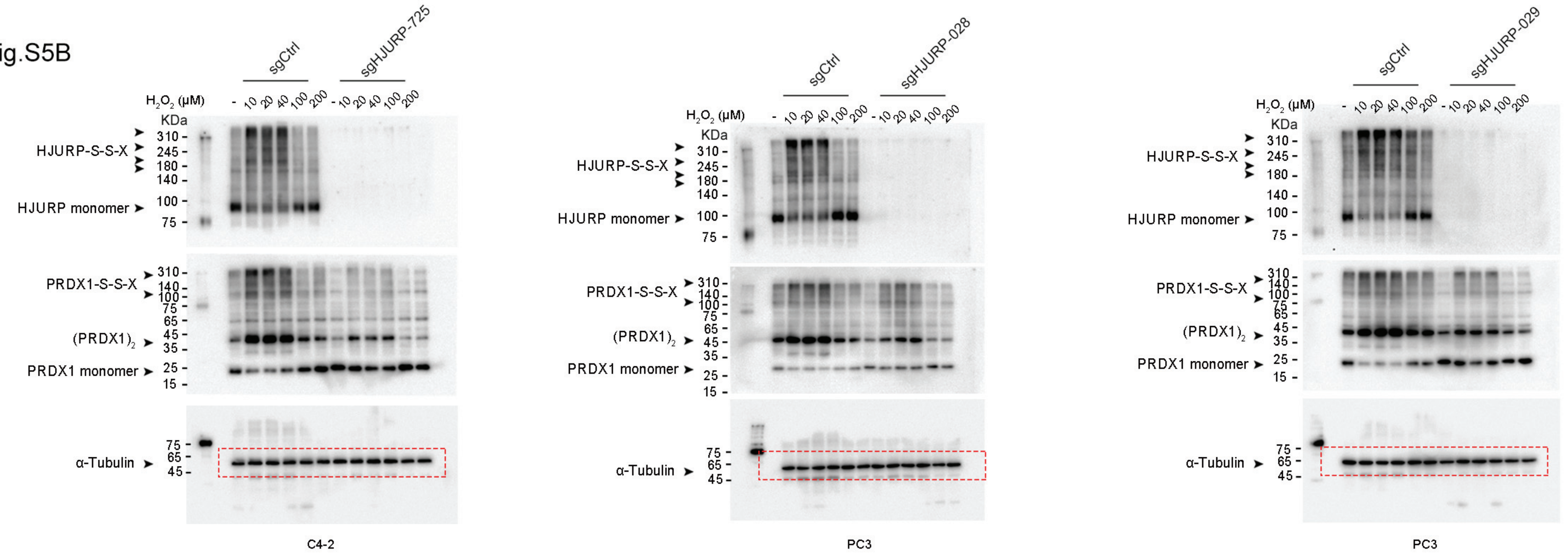

Fig.S5C

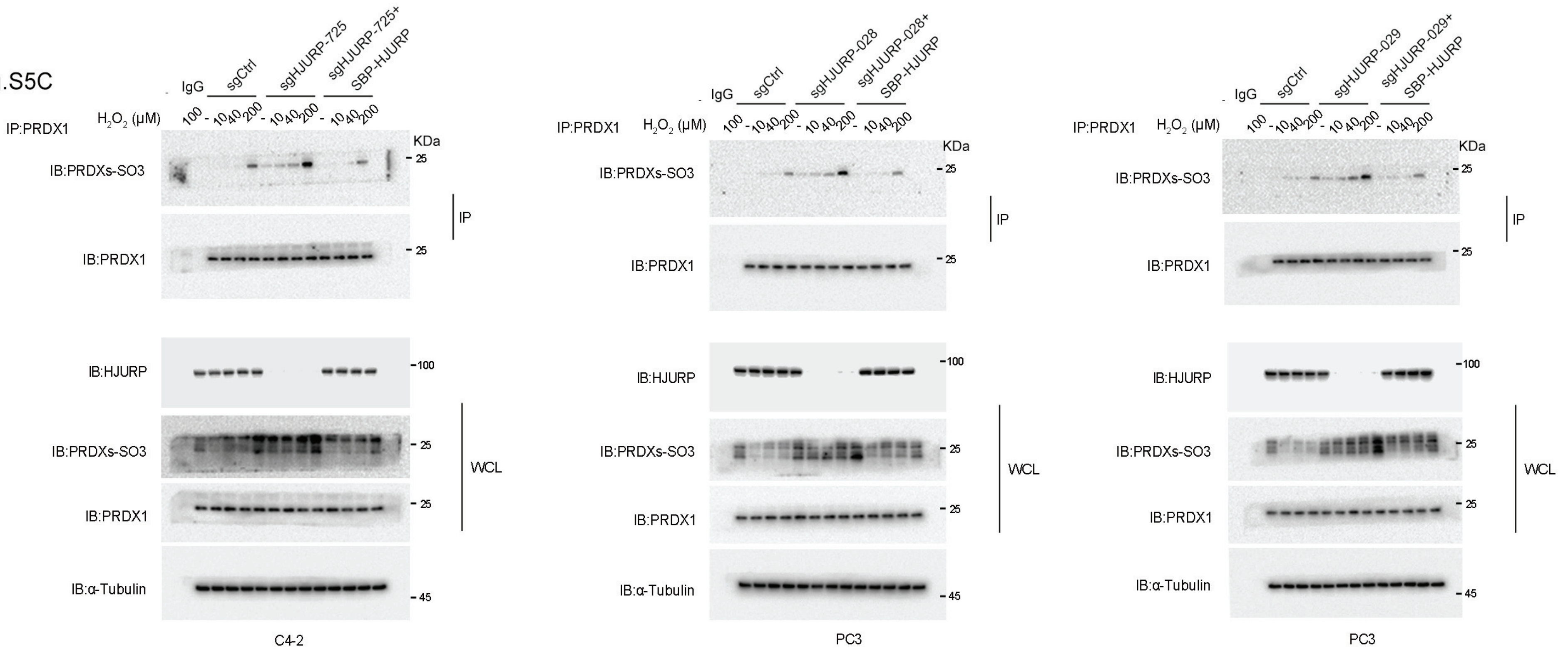

Fig.S5D

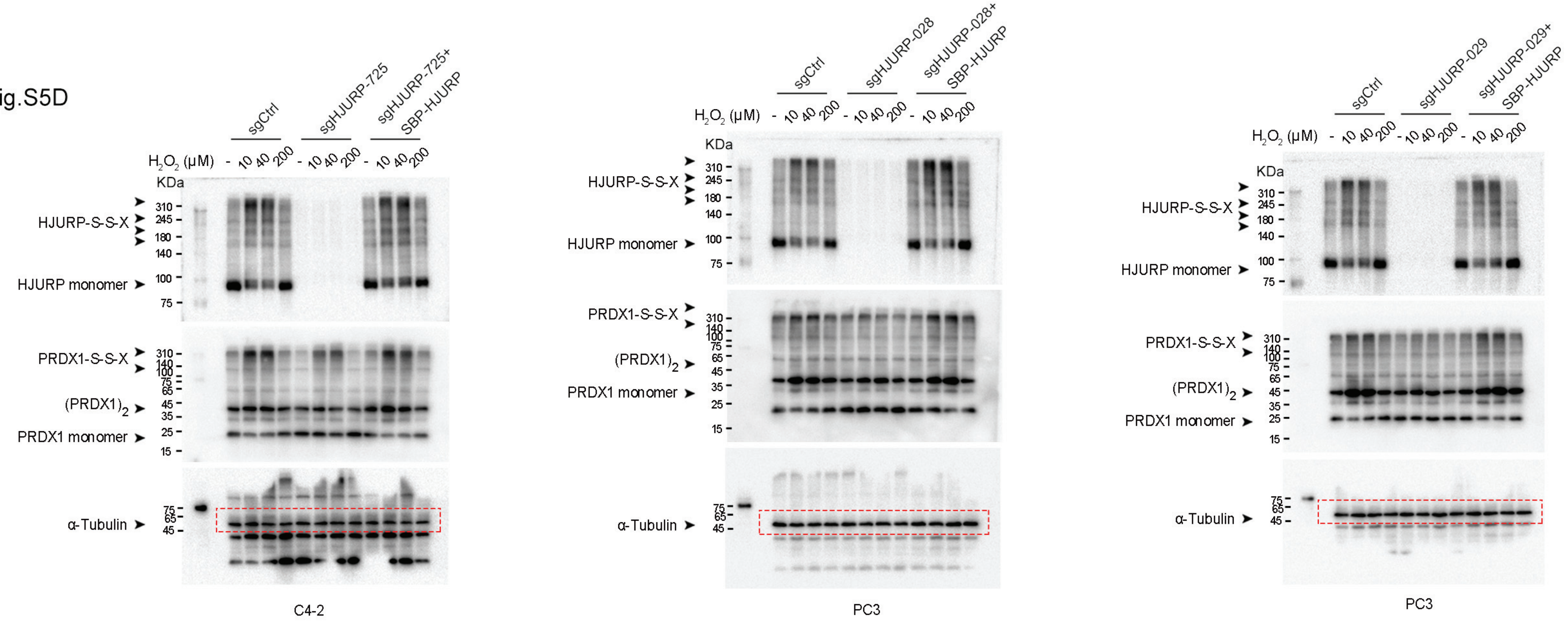

Fig.S5E

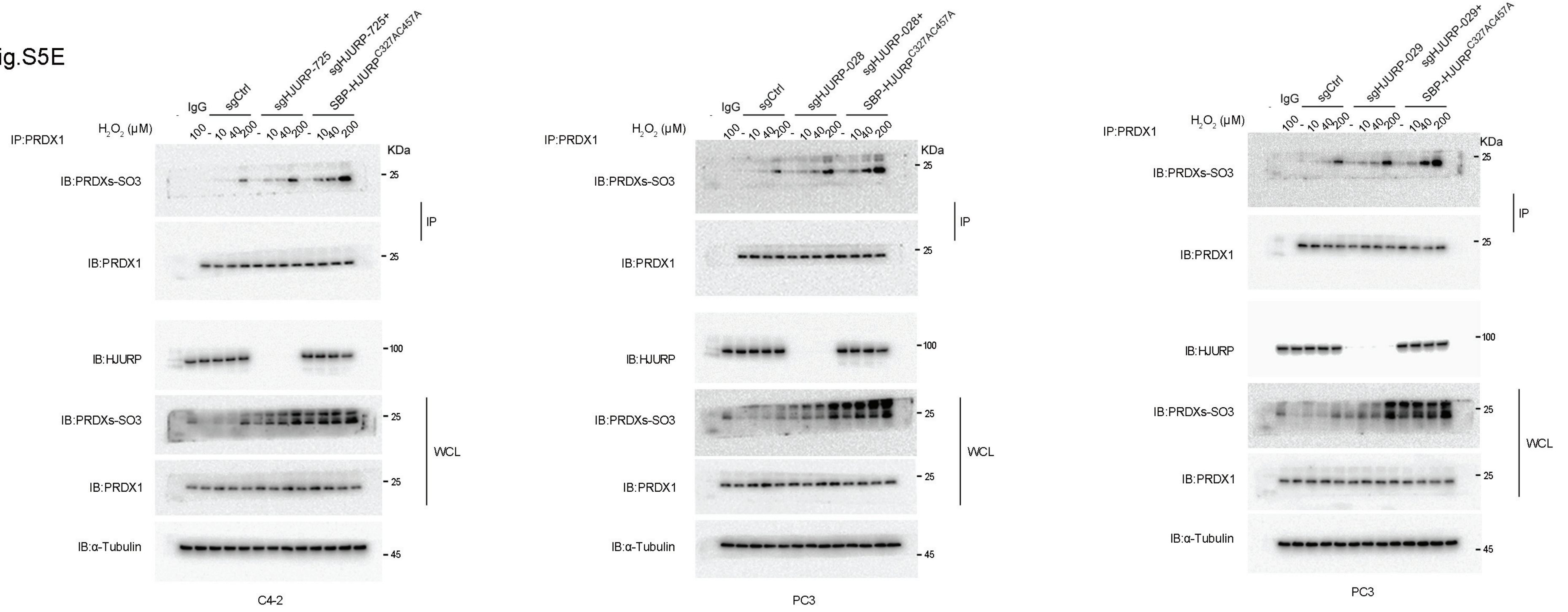

Fig.S5F

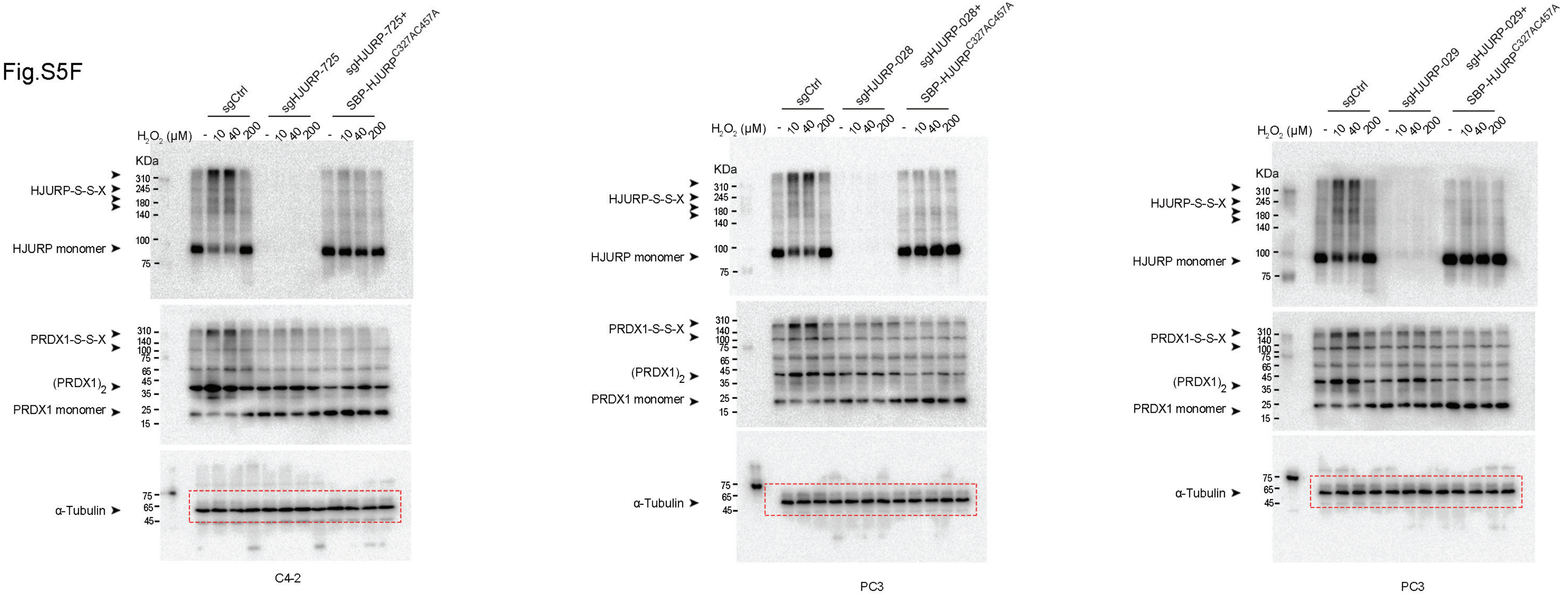

Fig.S6A

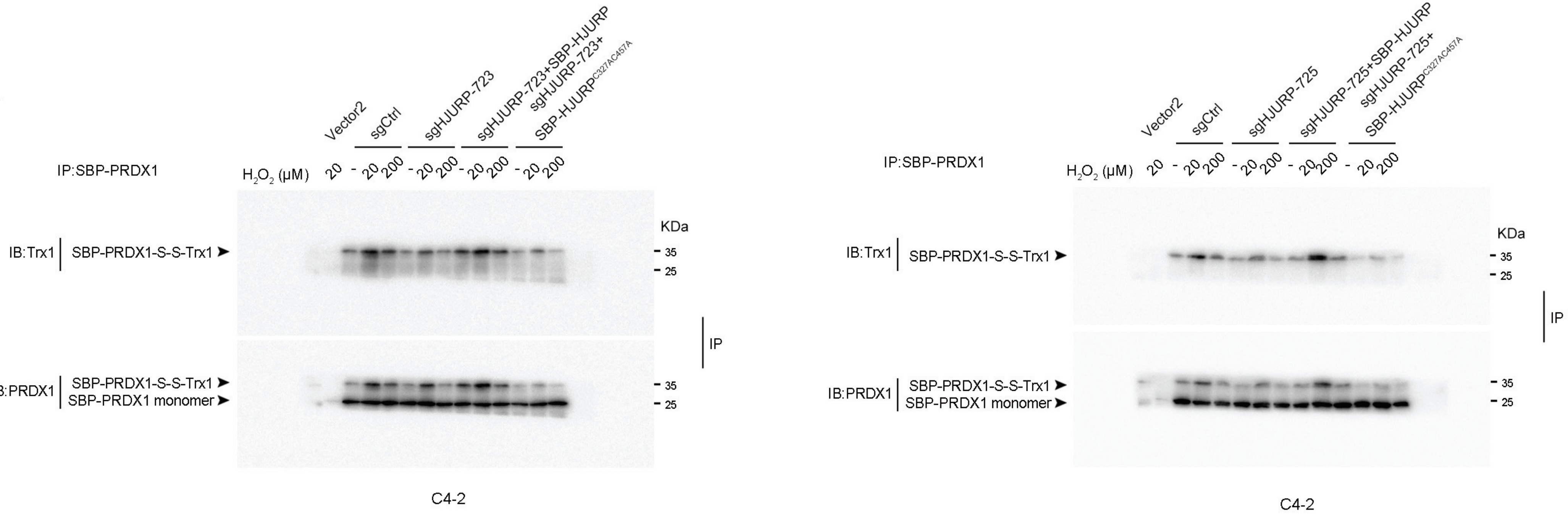

Fig.S6B

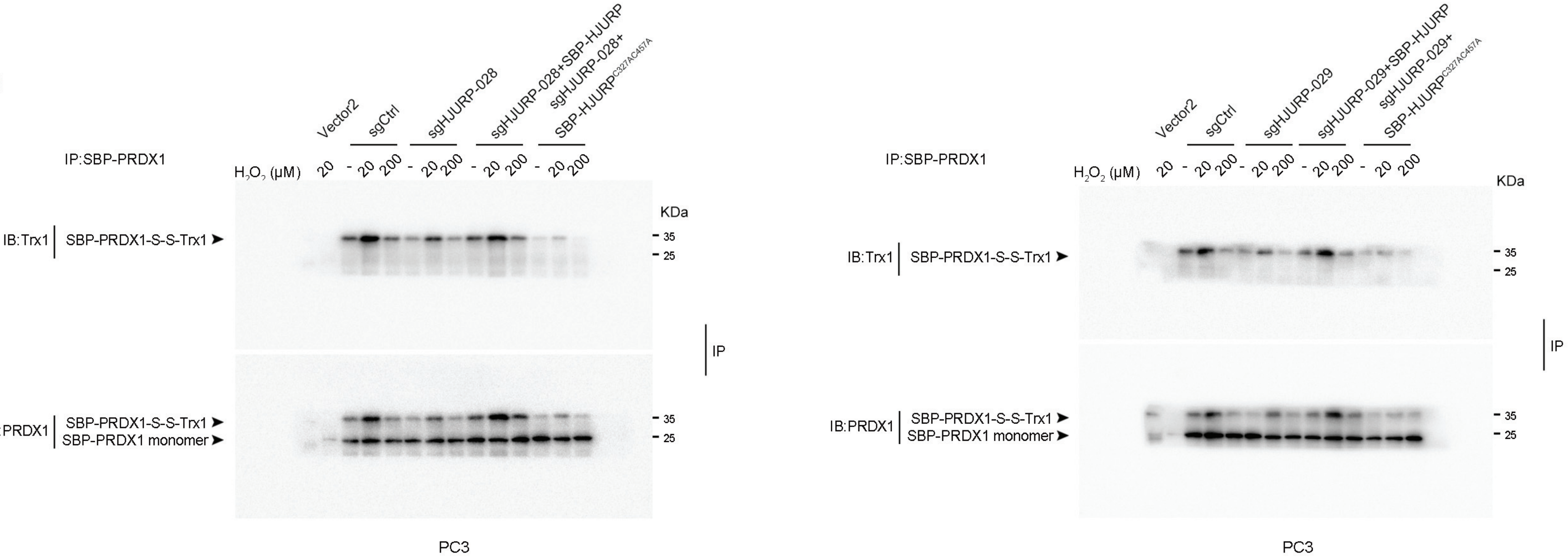

Fig.S6C

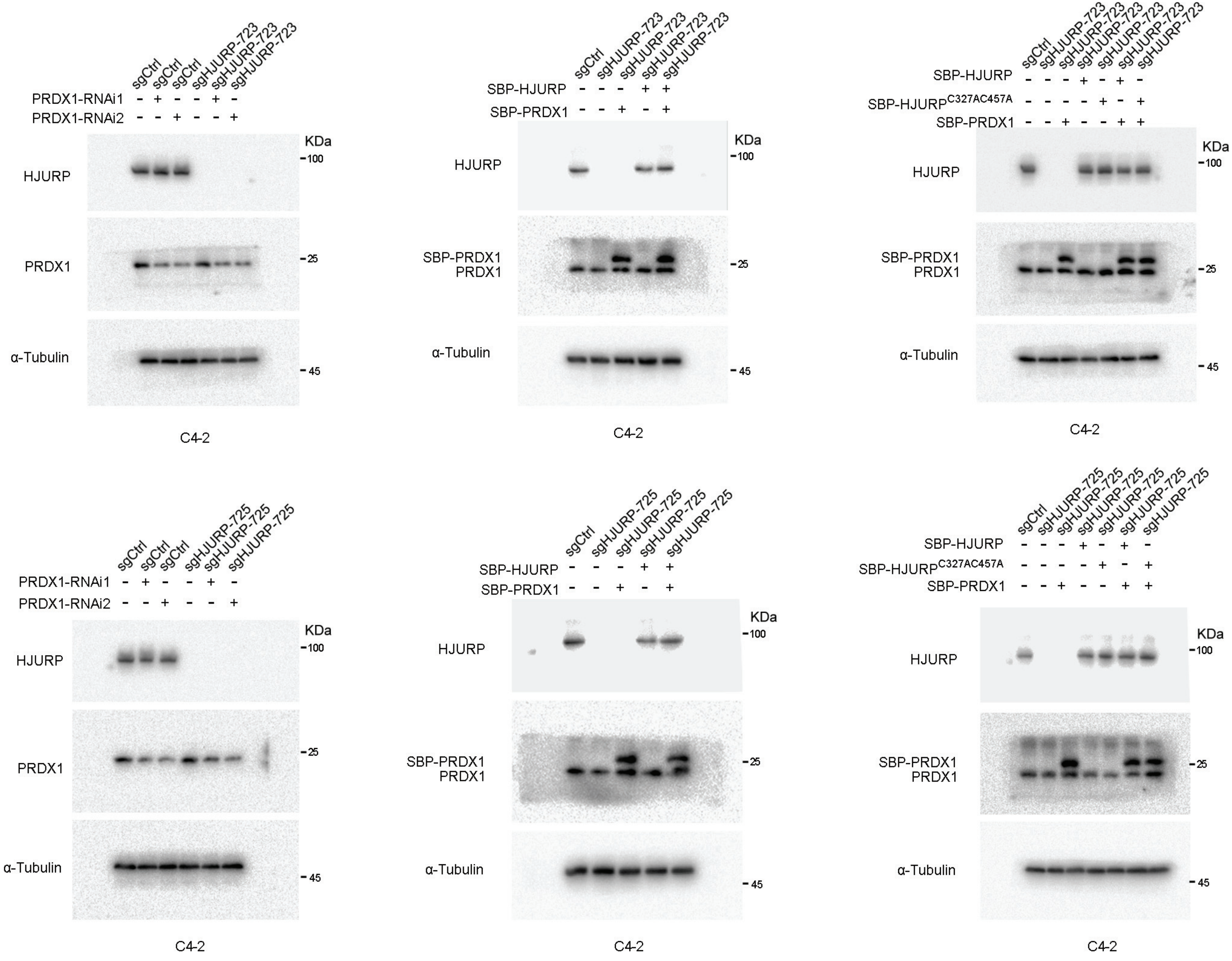
sgCtrl
sgCtrl
sgCtrl
sgHJURP-723
sgHJURP-723
sgHJURP-723
PRDX1-RNAi1 - + - - + -
PRDX1-RNAi2 - - + - - +
KDa
HJURP
PRDX1
α-Tubulin
100
25
45
C4-2
sgCtrl
sgHJURP-723
sgHJURP-723
sgHJURP-723
sgHJURP-723
SBP-HJURP - - - + +
SBP-PRDX1 - - + - +
KDa
HJURP
SBP-PRDX1
PRDX1
α-Tubulin
100
25
45
C4-2
sgCtrl
sgHJURP-723
sgHJURP-723
sgHJURP-723
sgHJURP-723
sgHJURP-723
sgHJURP-723
SBP-HJURP - - - + - + -
SBP-HJURPC327AC457A - - - - + - +
SBP-PRDX1 - - + - - + +
KDa
HJURP
SBP-PRDX1
PRDX1
α-Tubulin
100
25
45
C4-2
sgCtrl
sgCtrl
sgCtrl
sgHJURP-725
sgHJURP-725
sgHJURP-725
PRDX1-RNAi1 - + - - + -
PRDX1-RNAi2 - - + - - +
KDa
HJURP
PRDX1
α-Tubulin
100
25
45
C4-2
sgCtrl
sgHJURP-725
sgHJURP-725
sgHJURP-725
sgHJURP-725
SBP-HJURP - - - + +
SBP-PRDX1 - - + - +
KDa
HJURP
SBP-PRDX1
PRDX1
α-Tubulin
100
25
45
C4-2
sgCtrl
sgHJURP-725
sgHJURP-725
sgHJURP-725
sgHJURP-725
sgHJURP-725
sgHJURP-725
SBP-HJURP - - - + - + -
SBP-HJURPC327AC457A - - - - + - +
SBP-PRDX1 - - + - - + +
KDa
HJURP
SBP-PRDX1
PRDX1
α-Tubulin
100
25
45
C4-2

Fig.S6D

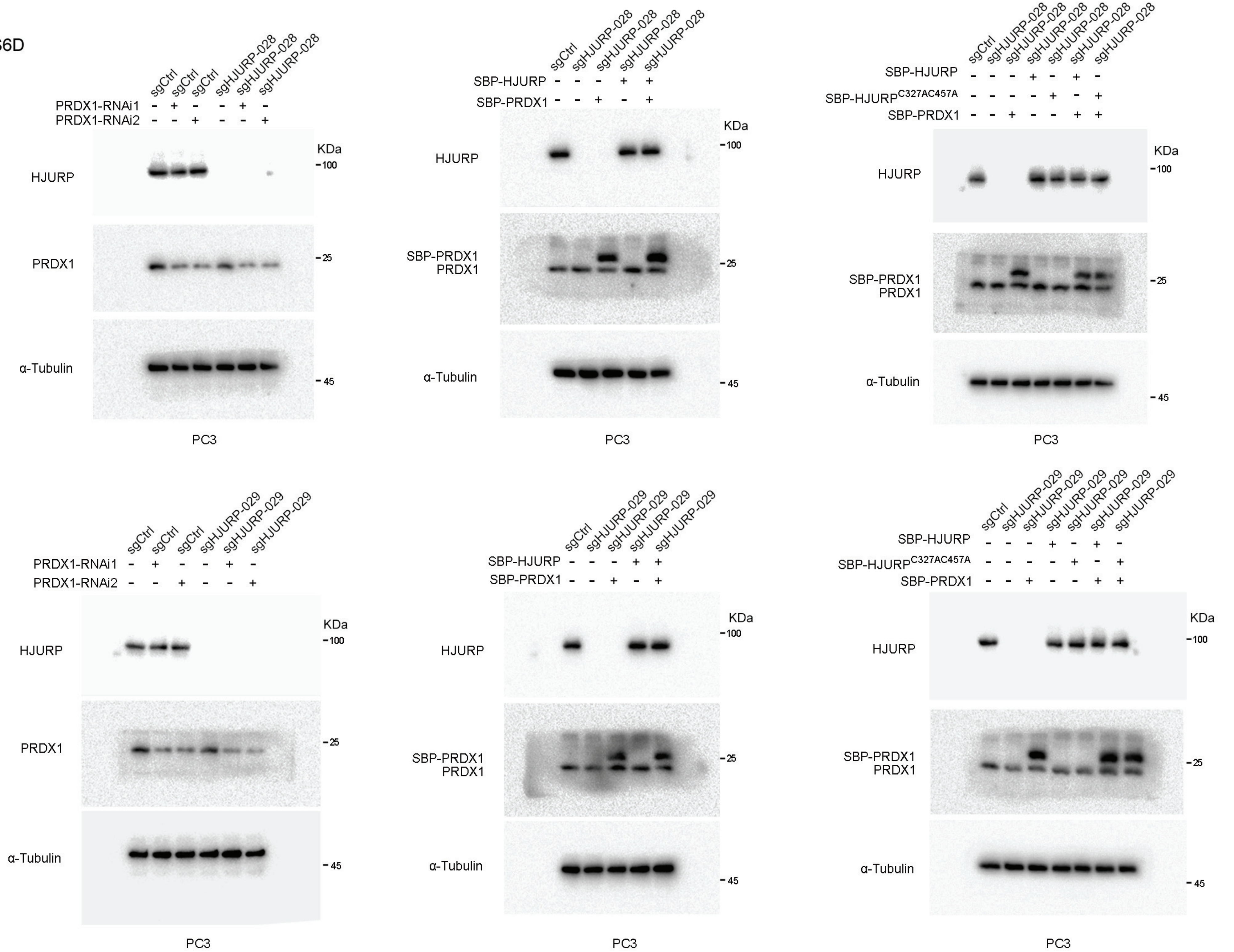
sgCtrl
sgHJURP-028
sgHJURP-029
PRDX1-RNAi1
PRDX1-RNAi2
SBP-HJURP
SBP-HJURP^{C327AC457A}
SBP-PRDX1
HJURP
PRDX1
SBP-PRDX1
α-Tubulin
KDa
100
25
45
PC3
