# Supplementary material for: HJURP inhibits sensitivity to ferroptosis inducers in prostate cancer cells by enhancing the peroxidase activity of PRDX1
Source: Redox Biol. 2024 Oct 10;77:103392. doi: 10.1016/j.redox.2024.103392 (PMC11525750; doi:10.1016/j.redox.2024.103392)
Supplement: Multimedia component 10 [file mmc10.docx]

| **Wild type** | **Forward Primer** | **Reverse Primer** |
| --- | --- | --- |
| HJURP | 5'-GGAATTCATGCTGGGTACGCTGCG-3' | 5'-ATAAGAATGCGGCCGCCACACTTTTAGTTTCCAATTTTTCT-3' |
| PRDX1 | 5'-CGGGATCCATGTCTTCAGGAAATGCTAAAAT-3' | 5'-ATAAGAATGCGGCCGCCTTCTGCTTGGAGAAATATTCTTTG-3' |
| **HJURP mutation** | **Forward Primer** | **Reverse Primer** |
| C166A | 5'-GTGCAGAGTATTTTGAGGCAGCAGGTAACAGAGCTG-3' | 5'-CAGCTCTGTTACCTGCTGCCTCAAAATACTCTGCAC-3‘ |
| C194A | 5'-GTGCCTGCCCCCGGATACGCAAGTCGTATCTCCAGAAAG-3' | 5'-CTTTCTGGAGATACGACTTGCGTATCCGGGGGCAGGCAC-3 |
| C256A | 5'-CTTTGAAGATGATGACATTGCCAATGTGACCATCAGTGAC-3' | 5'-GTCACTGATGGTCACATTGGCAATGTCATCATCTTCAAAG-3' |
| C310A | 5'-GGATGAACAAAACATATGCCAAAGGAGCCAGACGTTC-3' | 5'-GAACGTCTGGCTCCTTTGGCATATGTTTTGTTCATCC-3' |
| C327A | 5'-CAAGGAGAACTTCATACCCGCTTCTGAGCCTGTGAAAGGG-3' | 5'-CCCTTTCACAGGCTCAGAAGCGGGTATGAAGTTCTCCTTG-3' |
| C340A | 5'-GGACAGGGGCATTAAGAGATGCAAAGAACGTATTAGATGTTTC-3' | 5'-GAAACATCTAATACGTTCTTTGCATCTCTTAATGCCCCTGTCC-3' |
| C348A | 5'-CAAGAACGTATTAGATGTTTCTGCCCGTAAGACAGGTTTAAAATTG-3' | 5'-CAATTTTAAACCTGTCTTACGGGCAGAAACATCTAATACGTTCTTG-3' |
| C446A | 5'-CTTCATCGGGAATATGCCCTGAGTCCCAGGAAC-3' | 5'-GTTCCTGGGACTCAGGGCATATTCCCGATGAAG-3' |
| C457A | 5‘-GGAACCAGCCTCGCCGGATGGCACTCCCGGACTCCTGGGCCATG-3’ | 5‘-CATGGCCCAGGAGTCCGGGAGTGCCATCCGGCGAGGCTGGTTCC-3’ |
| C514A | 5'-CTCTGGAAGCAGGTAGGGCACTGCCCAAGAGCGATTC-3' | 5'-GAATCGCTCTTGGGCAGTGCCCTACCTGCTTCCAGAG-3' |
| C592A | 5'-CAAGCTTCATCAAAAGTATGCACTCAAATCTCCTGGGCAG-3' | 5'-CTGCCCAGGAGATTTGAGTGCATACTTTTGATGAAGCTTG-3' |
| C604A | 5'-GATGACAGTGCCTTTAGCTATTGGAGTGTCTACAG-3' | 5'-CTGTAGACACTCCAATAGCTAAAGGCACTGTCATC-3' |
| C646A | 5'-CATCACCCCTGGGGGCCAGAAAAAGTCTAC-3' | 5'-GTAGACTTTTTCTGGCCCCCAGGGGTGATG-3' |
| C663A | 5'-GCTCCTTCATCTACAGCCGTTGCTCGTGCCATC-3' | 5'-GATGGCACGAGCAACGGCTGTAGATGAAGGAGC-3' |
| **PRDX1 mutation** | **Forward Primer** | **Reverse Primer** |
| C52S | 5'-CTTCACCTTTGTGAGCCCCACGGAGATC-3' | 5'-GATCTCCGTGGGGCTCACAAAGGTGAAG-3' |
| C173S | 5'-CAAACATGGGGAAGTGAGTCCAGCTGGCTGGAAAC-3' | 5'-GTTTCCAGCCAGCTGGACTCACTTCCCCATGTTTG-3' |

**Table S1. Information of primers, target sequences, antibodies and reagents.**

|  | **Target sequence** |  |
| --- | --- | --- |
| PRDX1-RNAi1 | 5’-GGAGATCATTGCTTTCAGT-3’ | NA |
| PRDX1-RNAi2 | 5’-CAGCCTGTCTGACTACAAA-3’ | NA |

| **CRISPR-Cas9** | **sgRNA sequence** |  |
| --- | --- | --- |
| sgCtrl | 5’-CGCTTCCGCGGCCCGTTCAA-3’ | NA |
| sgHJURP-723 | 5’-TGTGACTCCGCTGCCTTCAC-3’ | NA |
| sgHJURP-725 | 5’-GCTGTCATTTCTAGGTACTA-3’ | NA |
| sgHJURP-028 | 5’-ACCATCAGTGACCTGTACGC-3’ | NA |
| sgHJURP-029 | 5’-TCTGTGGAGGAAGGATGCAA-3’ | NA |

| **Antibodies** | **Identifier** | **Source** |
| --- | --- | --- |
| HJURP | ab100800, ab233541 | Abcam, USA |
| PRDX1 | ab109498, ab109596 | Abcam, USA |
| IgG | ab6715 | Abcam, USA |
| α-Tubulin | ab176560 | Abcam, USA |
| PRDXs-SO_3_ | ab16830 | Abcam, USA |
| xCT^-^ | ab175186 | Abcam, USA |
| ACSL4 | ab155282 | Abcam, USA |
| GPX4 | ab125066 | Abcam, USA |
| PRDX2 | ab109367 | Abcam, USA |
| p-Ser/Thr | ab17464 | Abcam, USA |
| Acetyl-Lys | ab190479 | Abcam, USA |
| Ubiquitin | ab134953 | Abcam, USA |
| 4-HNE | ab48506 | Abcam, USA |
| Trx1 | ab133524 | Abcam, USA |

| **Reagent** | **Identifier** | **Source** |
| --- | --- | --- |
| Hydrogen peroxide solution (H_2_O_2_) | 323381 | Sigma, USA |
| Methyl methanethiosulfonate (MMTS) | 64306 | Sigma, USA |
| SA-Sepharose beads | SA10004 | Thermo Fisher, USA |
| Biotin | B20656 | Thermo Fisher, USA |
| DMSO | D2650 | Sigma, USA |
| RSL3 | SML2234 | Sigma, USA |
| Erastin | E7781 | Sigma, USA |
| PACMA31 | 5116 | Tocris, UK |
| Ferrostatin-1 | SML0583 | Sigma, USA |
| Liproxstatin-1 | SML1414 | Sigma, USA |
| Z-VD-FMK | 218744 | Sigma, USA |
| Necrosulfonamide | 480073 | Sigma, USA |
| 3-Methyladenine | M9281 | Sigma, USA |
| Docetaxel | 01885 | Sigma, USA |
| Rapamycin | 553210 | Sigma, USA |
| TNF-α | SRP3177 | Sigma, USA |
| Catalase | S0082 | Beyotime, China |
| Mercaptoethanol | 63689 | Sigma, USA |
| Triton X-100 | 93443 | Sigma, USA |
| Tris | B105639 | Aladdin, China |
| NaCl | C111533 | Aladdin, China |
| Protease inhibitor | A32953 | TargetMol, China |
| Phosphatase inhibitor | 78426 | Invitrogen, USA |
| Phenylmethanesulfonyl fluoride | P0100 | Solarbio, China |
