# Supplementary material for: HJURP inhibits sensitivity to ferroptosis inducers in prostate cancer cells by enhancing the peroxidase activity of PRDX1
Source: Redox Biol. 2024 Oct 10;77:103392. doi: 10.1016/j.redox.2024.103392 (PMC11525750; doi:10.1016/j.redox.2024.103392)
Supplement: Multimedia component 11 [file mmc11.docx]

**Table S2. Clinicopathological features of the study cohort.**

| **Characteristics**  All cases | **The Third Affiliated Hospital** | **Tissue microarray** |
| --- | --- | --- |
|  | 105 (100.0%) | 158 (100.0%) |
| Age in years, median (IQR) | 71 (66, 77) | 68.5 (64, 73) |
| Gleason grade group at RP |  |  |
| 1 | 23 (21.9%) | 12 (7.6%) |
| 2 | 23 (21.9%) | 47 (29.7%) |
| ≥3 | 59 (56.2%) | 99 (62.7%) |
| Pathological T stage |  |  |
| T2 | 43 (41.0%) | 130 (82.3%) |
| T3a | 23 (21.9%) | 22 (13.9%) |
| T3b | 37 (35.2%) | 6 (3.8%) |
| T4 | 2 (1.9%) | NA |
| Pathological N stage |  |  |
| Nx | 26 (24.8%) | NA |
| N0 | 51 (48.6%) | 151 (95.6%) |
| N1 | 28 (26.7%) | 7 (4.4%) |
| Surgical margins |  |  |
| Negative | 79 (75.2%) | 140 (88.6%) |
| Positive | 26 (24.8%) | 18 (11.4%) |

IQR, interquartile range; PSA, prostate-specific antigen; RP, radical prostatectomy; IRS, immunoreactivity score.
