# Supplementary material for: HJURP inhibits sensitivity to ferroptosis inducers in prostate cancer cells by enhancing the peroxidase activity of PRDX1
Source: Redox Biol. 2024 Oct 10;77:103392. doi: 10.1016/j.redox.2024.103392 (PMC11525750; doi:10.1016/j.redox.2024.103392)
Supplement: Multimedia component 12 [file mmc12.docx]

|  | **Univariable models** | | **Multivariable models** | | |  | **Multivariable models** | |
| --- | --- | --- | --- | --- | --- | --- | --- | --- |
| **Variables** | **HR (95%CI)** | ***P* value** |  | **HR (95%CI)** | ***P* value** |  | **HR (95%CI)** | ***P* value** |
| Age (years) |  |  |  |  |  |  |  |  |
| ≤65 | Ref. |  |  | Ref. |  |  | Ref. |  |
| >65 | 0.08 (0.01, 0.61) | 0.014 |  | 0.04 (0.00, 0.56) | 0.017 |  | 0.05 (0.00, 0.56) | 0.016 |
| Gleason grade group at RP |  |  |  | - |  |  | - |  |
| 1 | Ref. |  |  |  |  |  |  |  |
| 2 | 0.54 (0.05, 5.98) | 0.616 |  |  |  |  |  |  |
| ≥3 | 2.57 (0.34, 19.27) | 0.359 |  |  |  |  |  |  |
| Pathological T stage |  |  |  |  |  |  | - |  |
| T2 | Ref. |  |  | Ref. |  |  |  |  |
| T3a | 4.69 (1.58, 13.93) | 0.005 |  | 2.96 (0.73, 11.97) | 0.128 |  |  |  |
| T3b | 9.79 (3.04, 31.56) | <0.001 |  | 3.49 (0.82, 14.88) | 0.091 |  |  |  |
| Pathological N stage |  |  |  |  |  |  |  |  |
| N0 | Ref. |  |  | Ref. |  |  | Ref. |  |
| N1 | 14.98 (4.98, 45.08) | <0.001 |  | 13.35 (2.23, 79.81) | 0.005 |  | 21.80 (4.24, 111.96) | <0.001 |
| IRS of HJURP | 6.40 (3.69, 11.10) | <0.001 |  | 2.89 (1.10, 7.61) | 0.032 |  | 2.75 (1.07, 7.04) | 0.035 |
| IRS of PRDX1 | 4.80 (3.11, 7.42) | <0.001 |  | 3.94 (1.73, 8.98) | 0.001 |  | 3.35 (1.57, 7.18) | 0.002 |
| Surgical margins |  |  |  |  |  |  |  |  |
| Negative | Ref. |  |  | Ref. |  |  | Ref. |  |
| Positive | 5.77 (2.37, 14.03) | <0.001 |  | 11.44 (1.92, 68.01) | 0.007 |  | 11.56 (2.38, 56.19) | 0.002 |

**Table S3. Univariate and multivariate Cox regression analyses for overall survival in PCa patients enrolled in tissue microarray.**

IRS, immunoreactivity score; RP, radical prostatectomy
