# Supplementary material for: HJURP inhibits sensitivity to ferroptosis inducers in prostate cancer cells by enhancing the peroxidase activity of PRDX1
Source: Redox Biol. 2024 Oct 10;77:103392. doi: 10.1016/j.redox.2024.103392 (PMC11525750; doi:10.1016/j.redox.2024.103392)
Supplement: Multimedia component 13 [file mmc13.docx]

**Supplementary Figure Legends**

**Supplementary Fig. S1. Knockout of HJURP enhanced the sensitivity of PCa cells to ferroptosis inducers and inhibited migration and invasion without affecting docetaxel-induced apoptosis, rapamycin-induced autophagy, or TNF-α-induced necroptosis. A.** Knockout of HJURP in C4-2 and PC3 cells confirmed by western blotting and Sanger sequencing. **B-C.** HJURP knockout inhibited migration and invasion in C4-2 and PC3 cells. Wound width was normalized to sgCtrl cells at 0 hours. **D.** Cytotoxicity assay of HJURP knockout PC3 cells following treatment with indicated doses of Erastin or RSL3 for 24 hours. **E-G.** HJURP knockout C4-2 and PC3 cells were treated with docetaxel (**E**), rapamycin (**F**), or TNF-α (**G**) for 48 hours. **H.** HJURP expression was rescued in HJURP knockout C4-2 and PC3 cells. In **B** and **C**, unpaired 2-tailed *t* test was used to determine significance. In the rest of the figure, 1-way ANOVA test was used to determine significance. Error bars indicate the SD from three independent experiments. **P* < 0.05, ***P* < 0.01, ****P* < 0.001.

**Supplementary Fig. S2. HJURP inhibited PC3 cells sensitivity to ferroptosis inducers. A.** Cytotoxicity assay of HJURP rescued PC3 cells treated with indicated doses of Erastin or RSL3 for 24 hours. **B.** 24-hour dose-response curves of HJURP knockout PC3 cells with Erastin or RSL3 treatment. Relative cell viability was normalized to sgCtrl cells with Erastin (10^-2^ μM) or RSL3 (10^-2^ μM) treatment. **C.** 24-hour cytotoxicity assay of HJURP-regulated PC3 cells treated with Erastin (5 μM) or RSL3 (0.5 μM) in the absence or presence of ferrostatin-1 (2 μM), liproxstatin-1 (1 μM), Z-VAD-FMK (10 μM), necrosulfonamide (0.5 μM), or 3-methyladenine (250 μM). **D-F.** Clonogenic survival assays (Erastin, 1 μM, 12 days; RSL3, 80 nM, 12 days; Ferrostatin-1, 1 μM, 12 days; **D**), lipid peroxidation (Erastin, 5 μM, 8 hours; RSL3, 1 μM, 2 hours; Ferrostatin-1, 2 μM, 24 hours; **E**) and MDA production (Erastin, 5 μM, 8 hours; RSL3, 1 μM, 2 hours; Ferrostatin-1, 2 μM, 24 hours; **F**) in PC3 cells treated with/without Erastin, RSL3, or ferrostatin-1. 1-way ANOVA test was used to determine significance. The colony number of the RSL3/Erastin or RSL3/Erastin+Ferrostatin-1 group in each HJURP-regulated group (sgCtrl. sgHJURP, etc.) was normalized to the colony number of its own Ctrl group. Colony inhibition (%) = 1- [colony number (RSL3 / Erastin or RSL3 / Erastin+Ferrostatin-1) / colony number (Ctrl)]×100%. Error bars indicate the SD from three independent experiments (Error bars for clonogenic survival assays were calculated from two independent experiments). **P* < 0.05, ***P* < 0.01, ****P* < 0.001. IC_50_, half maximal inhibitory concentration.

**Supplementary Fig. S3. HJURP inhibited sensitivity to ferroptosis inducers via the PRDX1/ROS pathway.** **A.** HJURP did not affect the protein levels of xCT^-^, ACSL4, or GPX4. **B-C.** Fe^2+^ (**B**) and GSH (**C**) assays in HJURP-regulated C4-2 or PC3 cells. RSL3, 1 μM, 2 hours; Erastin, 5 μM, 10 hours; NAC, 5 mM, 24 hours; CH, 50 μM, 24 hours. **D.** LC-MS/MS analysis revealed that PRDX1 or PRDX2 was the partner protein of HJURP. **E.** Co-IP showed that HJURP resided in the same complex with PRDX1 but not PRDX2. **F-G.** HJURP suppressed intracellular ROS production by a PRDX1-dependent pathway in PC3 cells. **H-K.** Cytotoxicity assay (RSL3, 0.5 μM, 24 hours; Ferrostatin-1, 2 μM, 24 hours; **H**), Clonogenic survival assays (RSL3, 80 nM, 12 days; **I**), lipid peroxidation (RSL3, 1 μM, 2 hours; Ferrostatin-1, 2 μM, 24 hours; **J**) and MDA production (RSL3, 1 μM, 2 hours; Ferrostatin-1, 2 μM, 24 hours; **K**) assays in HJURP-regulated PC3 cells. **L-M.** CCK8 assay of C4-2 or PC3 cells following treatment with indicated doses of H_2_O_2_ for 24 hours. In **G**, RSL3 group of **H**, **I**, **J**, **K** and 200, 400 group of **L**, **M**, unpaired 2-tailed *t* test was used to analyze the differences between sgCtrl and sgHJURP or sgHJURP and sgHJURP+HJURP-flag respectively. In the rest of the figure, 1-way ANOVA test was used to determine significance. The colony number of the RSL3 group in each HJURP-regulated group (sgCtrl. sgHJURP, etc.) was normalized to the colony number of its own Ctrl group. Colony inhibition (%) = 1-[colony number (RSL3) / colony number (Ctrl)]×100%. Error bars indicate the SD from three independent experiments (Error bars for clonogenic survival assays were calculated from two independent experiments). **P* < 0.05, ***P* < 0.01, ****P* < 0.001. NAC, N-acetyl-L-cysteine; CH, Catechin hydrate.

**Supplementary Fig. S4. HJURP formed disulfide-linked intermediates with PRDX1. A-D.** HJURP did not affect the expression (**A**), phosphorylation (**B**), acetylation (**C**), or ubiquitination (**D**) of PRDX1 in C4-2 and PC3 cells. **E.** Non-reducing and reducing immunoblotting of HJURP and PRDX1 in PC3 cells treated with indicated H_2_O_2_ concentrations for 2 minutes. **F-G.** Interfering with PRDX1 expression (**F**) or mutating Cys^52^ and Cys^173^ of PRDX1 (**G**) inhibited the formation of HJURP-S-S-X in C4-2 and PC3 cells. **H.** SBP-HJURP and SBP-PRDX1 were purified using SA-based affinity purification, and these purified proteins were next subjected to non-reducing immunoblotting.

**Supplementary Fig. S5. HJURP enhanced the peroxidase activity of PRDX1 via disulfide binding. A-D.** HJURP knockout promoted hyperoxidation of PRDX1 (**A**) while inhibiting the formation of PRDX1 disulfide-linked conjugates (**B**) in C4-2 and PC3 cells following treatment with indicated H_2_O_2_ concentrations for 2 minutes; however, rescuing HJURP expression reversed that (**C-D**). **E-F.** Mutating both Cys^327^ and Cys^457^ of HJURP led to increased PRDX1 hyperoxidation (**E**) but less formation of PRDX1 disulfide-linked conjugates (**F**).

**Supplementary Fig. S6. Immunoblot analysis of PRDX1-S-S-Trx1, HJURP and PRDX1 in C4-2 and PC3 cells with indicated treatment. A-B.** Non-reducing western blotting showed that HJURP, but not HJURP^C327AC457A^, promoted PRDX1-S-S-Trx1 formation in C4-2 (**A**) and PC3 (**B**) cells. **C-D.** Western blotting confirmed the expression of HJURP and PRDX1 in C4-2 (**C**) and PC3 (**D**) cells transfected with SBP-HJURP, SBP-PRDX1, PRDX1-RNAi, or SBP-HJURP^C327AC457A^.

**Supplementary Fig. S7. HJURP cooperated with PRDX1 to inhibit the sensitivity to ferroptosis inducers in PCa cells.** **A-C.** HJURP, but not HJURP^C327AC457A^, synergized with PRDX1 to suppress cell death (**A**) induced by RSL3 (1 μM for C4-2 cells and 0.5 μM for PC3 cells, 24 hours), scavenge intracellular ROS (**B**) or promoted colony formation of PCa cells with RSL3 treatment (C4-2, 250 nM, 12 days; PC3, 80 nM, 12 days; **C**). 1-way ANOVA test was used to analyze the differences between sgCtrl and PRDX1-RNAi or sgHJURP and PRDX1-RNAi+sgHJURP, respectively. In the rest of the figure, unpaired 2-tailed *t* test was used to determine significance. The colony number of the RSL3 group in each HJURP-regulated group (sgCtrl. sgHJURP, PRDX1-RNAi, etc.) was normalized to the colony number of its own Ctrl group. Colony inhibition (%) = 1-[colony number (RSL3) / colony number (Ctrl)]×100%. Error bars indicate the SD from three independent experiments (Error bars for clonogenic survival assays were calculated from two independent experiments). **P* < 0.05, ***P* < 0.01, ****P* < 0.001.

**Supplementary Fig. S8.** **HJURP synergized with PRDX1 to protect PCa cells from lipid peroxidation induced by RSL3.** **A-C.** HJURP, but not HJURP^C327AC457A^, synergized with PRDX1 to inhibit MDA production (RSL3, 1 μM, 2 hours) in C4-2 and PC3 cells. **D-F.** C4-2 (**D**) and PC3 (**E-F**) cells treated with or without RSL3 (1 μM, 2 hours) were stained with BODIPY 581/591 C11 for flow cytometry analysis. 1-way ANOVA test was used to analyze the differences between sgCtrl and PRDX1-RNAi or sgHJURP and PRDX1-RNAi+sgHJURP, respectively. In the rest of the figure, unpaired 2-tailed *t* test was used to determine significance. Error bars indicate the SD from three independent experiments. **P* < 0.05, ***P* < 0.01, ****P* < 0.001.
